# Different Shades of *Listeria monocytogenes*: Strain, Serotype, and Lineage-Based Variability in Virulence and Stress Tolerance Profiles

**DOI:** 10.3389/fmicb.2021.792162

**Published:** 2022-01-04

**Authors:** Francis Muchaamba, Athmanya K. Eshwar, Marc J. A. Stevens, Roger Stephan, Taurai Tasara

**Affiliations:** Institute for Food Safety and Hygiene, Vetsuisse Faculty, University of Zürich, Zurich, Switzerland

**Keywords:** *Listeria monocytogenes*, virulence, stress, genome, zebrafish, lysozyme

## Abstract

*Listeria monocytogenes* is a public health and food safety challenge due to its virulence and natural stress resistance phenotypes. The variable distribution of *L. monocytogenes* molecular subtypes with respect to food products and processing environments and among human and animal clinical listeriosis cases is observed. Sixty-two clinical and food-associated *L. monocytogenes* isolates were examined through phenome and genome analysis. Virulence assessed using a zebrafish infection model revealed serotype and genotype-specific differences in pathogenicity. Strains of genetic lineage I serotype 4b and multilocus sequence type clonal complexes CC1, CC2, CC4, and CC6 grew and survived better and were more virulent than serotype 1/2a and 1/2c lineage II, CC8, and CC9 strains. Hemolysis, phospholipase activity, and lysozyme tolerance profiles were associated with the differences observed in virulence. Osmotic stress resistance evaluation revealed serotype 4b lineage I CC2 and CC4 strains as more osmotolerant, whereas serotype 1/2c lineage II CC9 strains were more osmo-sensitive than others. Variable tolerance to the widely used quaternary ammonium compound benzalkonium chloride (BC) was observed. Some outbreak and sporadic clinical case associated strains demonstrated BC tolerance, which might have contributed to their survival and transition in the food-processing environment facilitating food product contamination and ultimately outbreaks or sporadic listeriosis cases. Genome comparison uncovered various moderate differences in virulence and stress associated genes between the strains indicating that these differences in addition to gene expression regulation variations might largely be responsible for the observed virulence and stress sensitivity phenotypic differences. Overall, our study uncovered strain and genotype-dependent variation in virulence and stress resilience among clinical and food-associated *L. monocytogenes* isolates with potential public health risk implications. The extensive genome and phenotypic data generated provide a basis for developing improved *Listeria* control strategies and policies.

## Introduction

*Listeria monocytogenes* is an important foodborne pathogen that accounts for serious public health problems and food safety challenges by causing severe clinical illnesses and high mortality in vulnerable human populations (European Food Safety Authority [EFSA], 2017; Centers for Disease Control [CDC], 2018; Radoshevich and Cossart, 2018). Infections usually arise from the consumption of contaminated food and can manifest as life-threatening meningitis, bacteremia, and feto-maternal complications (Allerberger and Wagner, 2010; European Food Safety Authority [EFSA], 2017; Radoshevich and Cossart, 2018). *L. monocytogenes* is genetically diverse, comprising 14 serotypes, four main evolutionary genetic lineages, and numerous multilocus sequence types (MLSTs). Recently, an *L. monocytogenes* serotype 4 h hybrid sub-lineage II comprising of hypervirulent strains was reported (Yin et al., 2019). Despite all *L. monocytogenes* strains being considered equally virulent by most food safety authorities worldwide, molecular epidemiological evidence shows the variable distribution of the different *L. monocytogenes* genetic and serological subtypes with respect to food products and processing environment as well as among human and animal clinical listeriosis cases (Maury et al., 2016; Fritsch et al., 2018). Serotypes 1/2a, 1/2b, 1/2c, and 4b are overrepresented in food and clinical isolates, with serotype 4b making up the bulk of human listeriosis cases (Ward et al., 2008; Orsi et al., 2011). Moreover, MLST unraveled clonal structure (Ragon et al., 2008) that shows an uneven distribution of clonal complexes (CCs) in clinical and food isolates leading to the identification of some hyper- and hypovirulent clones (Maury et al., 2016; Painset et al., 2019). Increased pathogenicity in some genetic backgrounds of *L. monocytogenes* has been demonstrated using mouse and *Galleria mellonella*-based virulence models (Maury et al., 2016; Lee et al., 2019). In addition, we recently demonstrated virulence variability among listeriosis outbreak strains representing different serotypes and genetic backgrounds (Muchaamba et al., 2019). *L. monocytogenes* has high genetic conservation, and the genetic background-associated difference in pathogenicity has been suggested to be mainly due to variable gene expression in the host between the different subgroups (Lee et al., 2019; Muchaamba et al., 2019). However, the recently described serotype 4h strains hypervirulence is attributed to an extra truncated *Listeria* pathogenicity islands (LIPI)-2 locus and unique cell wall teichoic acid (WTA) structure (Yin et al., 2019). In contrast, additional LIPIs such as LIPI-3 and LIPI-4 among some lineage I (LI) strains also contribute to virulence differences (Maury et al., 2016). Considering all *L. monocytogenes* to be equally pathogenic carries an inherent problem of underestimating and overestimating the risks posed by the hypervirulent and avirulent strains, respectively. This has the potential of declaring food contaminated with low numbers of hypervirulent strains to be safe when not and can lead to recalls and condemnation of otherwise safe to eat food contaminated with avirulent strains.

The current challenges posed by *L. monocytogenes* to public health and food safety are down to its natural stress resilience and virulence (Gandhi and Chikindas, 2007; Liu et al., 2007; Cossart, 2011; Bucur et al., 2018). *L. monocytogenes* has evolved various regulatory protein systems that control the necessary stress adaption and virulence responses, which allow stress survival and transmission along the food chain and subsequent host infection and pathogenicity. Stress resistance mechanisms in this bacterium include gene expression regulating proteins such as alternative sigma factors and cold shock domain family proteins (Csps) (O’Byrne and Karatzas, 2008; Schmid et al., 2009; Radoshevich and Cossart, 2018; Muchaamba et al., 2021a).

Several hurdle techniques, including acid, osmotic stress, antimicrobial peptides, desiccation, and disinfectants, are used to reduce the growth, survival, and persistence of *L. monocytogenes* in food and food-processing environments (Bucur et al., 2018; Wiktorczyk-Kapischke et al., 2021). In response, *L. monocytogenes* deploys a plethora of response mechanisms such as transporters, efflux pumps, and biofilm formation to facilitate the nutrient acquisition and utilization, stress tolerance, and persistence on food and in the environment (Gandhi and Chikindas, 2007; Ferreira et al., 2014; Buchanan et al., 2017; Stoller et al., 2019; Kragh et al., 2020). Some of these mechanisms have genetic background-based distribution resulting in phenotypic variability among *L. monocytogenes* strains (Hingston et al., 2017; Muchaamba et al., 2019; Wambui et al., 2020; Gelbicova et al., 2021). For instance, stress survival islet 2 and *qacH_*Tn*6188*-encoding benzalkonium chloride (BC) resistance predominantly occur in CC121 (Harter et al., 2017; Gelbicova et al., 2021). In contrast, the allose utilization cassette only occurs in lineage II (LII) strains (Liu et al., 2017; Muchaamba et al., 2019). Single-nucleotide polymorphisms (SNPs) also contribute to such differences. For example, isolates with full-length HtrA protease show higher virulence and stress tolerance relative to those harboring a premature stop codon (PMSC) in this gene (Stack et al., 2005; Wilson et al., 2006; Muchaamba et al., 2020). In contrast, strains carrying an L461T mutation in the pore-forming cytolysin, listeriolysin O (LLO), display significantly reduced virulence (Glomski et al., 2002). Collectively, these differences contribute to genetic background-associated differences in *L. monocytogenes* virulence and tolerance to food preservatives and cleaning and disinfectant chemicals, including salt, nisin, and quaternary ammonium compounds (Bergholz et al., 2010; Meier et al., 2017; Wambui et al., 2020). Furthermore, these factors and differences in nutrient acquisition and utilization capacities result in variability in the survival and growth of *L. monocytogenes* strains on food and in food-processing environments (Muchaamba et al., 2019). Therefore, generalization of findings of growth and survival potential using a strain from one genetic background might not be appropriate and could result in underestimation or overestimation of risk (Fritsch et al., 2018).

*L. monocytogenes* host virulence depends on its ability to evade immune defenses and hijack host cell systems and machinery, promoting its intracellular life cycle (Camejo et al., 2011; Cossart, 2011; Zhang et al., 2017; Radoshevich and Cossart, 2018). Infection onset involves the expression of virulence genes clustered in pathogenicity islands, some of which have phylogenetically distinctive distribution (Glaser et al., 2001; Vázquez-Boland et al., 2001; Clayton et al., 2014; Maury et al., 2016). Some strains have additional virulence determinants, such as additional pathogenicity islands (Nelson et al., 2004; den Bakker et al., 2012; Maury et al., 2016). *L. monocytogenes* also utilizes a host of strategies to evade the immune system, including enzymes encoded by the *dltABCD*, *mprF*, *pgdA* (peptidoglycan *N*-deacetylase), *gltC*, and *rmlT* (rhamnosyltransferase) genes, which are involved in cell wall modifications that increase tolerance to lysozyme and host defense antimicrobial peptides (Carvalho et al., 2014, 2015). Moreover, several small non-coding RNAs have direct and indirect involvement in fine-tuning virulence determining systems (Toledo-Arana et al., 2009; Bécavin et al., 2014; Cerutti et al., 2017; Lebreton and Cossart, 2017). Most of *L. monocytogenes* virulence factors are primarily regulated through the transcription regulator PrfA, which is also controlled through stress response regulatory proteins such as sigma factors and Csps (Nadon et al., 2002; Kazmierczak et al., 2003; de las Heras et al., 2011; Eshwar et al., 2017; Gaballa et al., 2019; Muchaamba et al., 2021a). AgrA, the response regulator of the Agr system, LysR-type transcriptional regulators, and several non-coding RNAs are also part of this regulatory network (Autret et al., 2003; Lebreton and Cossart, 2017; Abdelhamed et al., 2020).

Based on genomic analysis, a range of molecular indicators such as the detection of point mutations or PMSCs in *hly*, *prfA*, *inlA*, and *inlJ* were proposed as virulence potential estimates of *L. monocytogenes* isolates (Olier et al., 2003; Rousseaux et al., 2004; Liu et al., 2007; Port and Freitag, 2007; Van Stelten et al., 2010; Maury et al., 2017). Furthermore, several studies have suggested that differences in conserved gene expression between hypervirulent and hypovirulent isolates in the early phases of infection are key for infection progression and could reflect the contribution of regulatory networks to fine-tuning virulence (Severino et al., 2007; Wurtzel et al., 2012; Gahan and Hill, 2014; Lee et al., 2019). Our ability to control the foodborne transmission of *L. monocytogenes* and mitigate the impacts of its infection in humans and animals thus requires an improved understanding of such molecular mechanisms involved in the expression regulation of its stress resistance and virulence phenotypes.

The variable pathogenic potential of *L. monocytogenes* is further compounded because no known infectious dose of this bacterium has yet been determined. This means that the allowable number of 100 CFU per gram of some ready-to-eat (RTE) food said not to support the growth of *Listeria* spp. might be infectious if contaminated by a hypervirulent strain. This acceptable limit has been arrived at by considering all *L. monocytogenes* strains to be equally virulent, have the same infectious dose, and have similar growth capacities on food (Fritsch et al., 2018; Rahman et al., 2018). Moreover, it does not consider disease dynamics where host factors and environmental factors play a role in disease outcomes. Therefore, this acceptable limit might represent a far more than enough infectious dose for some sections of the population. It is with this in mind that we set out to investigate the virulence potential and stress tolerance profiles of strains representing different *L. monocytogenes* genetic backgrounds. A collection of 62 clinical- and food-associated *L. monocytogenes* isolates were examined through phenome and genome analyses. We aimed to determine if there might be links between genetic background, stress resistance, and virulence that could explain the variable distribution of different *L. monocytogenes* strains with respect to food products and processing environment and among human and animal clinical listeriosis cases.

## Materials and Methods

### Ethics Statement

All protocols used in this study adhered to the standards of the “Ordinance on laboratory animal husbandry, the production of genetically modified animals and the methods of animal experimentation; Animal Experimentation Ordinance” (SR 455.163, April 12, 2010), Swiss Federal Food Safety and Veterinary Office (FSVO/BLV). The maximum age of the embryos during experimentation was 5 days post-fertilization; therefore, no license was required from the cantonal veterinary office in Switzerland, as such embryos will not have yet reached the free-feeding stage. Husbandry and breeding of the adult zebrafishes were performed under the supervision of Prof. Stephan Neuhauss, Institute for Molecular Life Sciences, University of Zürich, Zurich, Switzerland. Internationally recognized standards and Swiss legal, ethical guidelines for the use of fish in biomedical research were followed for all animal protocols. The local authorities approved all the experiments (Veterinäramt Zürich Tierhaltungsnummer 150).

### Bacterial Strains and Culture Conditions

*Listeria monocytogenes* isolates used in this study are listed in Supplementary Table 1. Bacteria were stored at −80°C in brain heart infusion medium (BHI, Oxoid, United Kingdom) supplemented with 20% glycerol. Strains were initially grown overnight on blood agar or BHI agar plates at 37°C to get single colonies, and then pre-cultured twice in 10-ml BHI broth (37°C, 150 rpm) for 16 h, to get stationary phase secondary cultures. Unless otherwise stated, such secondary BHI pre-cultures were used for experiments.

### Zebrafish Microinjection Assays

The zebrafish embryo-based infection model was used to compare *in vivo* virulence profiles between the study strains. Assays were performed using the *Danio rerio wik* zebrafish line strains as previously described (Eshwar et al., 2017). Briefly, bacteria for microinjection experiments were harvested from secondary stationary phase stage BHI cultures prepared as described earlier by centrifugation (5,000 × *g* for 10 min), washed once, and diluted to 5 × 10^8^ CFU ml^–1^ in Dulbecco’s phosphate-buffered saline. Two-day post-fertilization embryos (*n* = 10 per strain) were infected with different *L. monocytogenes* strains (500 CFU) *via* the caudal vein. Post-infection, a single embryo was placed into a well of a 24-well plate containing 1-ml E3 medium. The embryos were incubated at 28°C and monitored twice a day under a stereomicroscope for signs of disease and eventual death until 72-h post-infection (hpi). The injected CFU numbers were confirmed through viable cell counting performed on five individual embryos disintegrated immediately after microinjection. Additionally, bacterial growth profiles of selected strains representing genetic lineage LI, LII, and lineage III (LIII) (highlighted in Supplementary Table 1) were assessed by periodically determining the CFU of each strain in the embryos. Viable cell counts were performed on five individual embryos per strain disintegrated at 0, 16, 24, and 48 hpi. The lysates from these embryos were serial diluted and plated out on Agar *Listeria* according to Ottaviani and Agosti plates, then incubated for 24 h at 37°C. For all experiments, at least three independent biological repeats were done.

### Hemolysis and Phosphatidylinositol-Specific Phospholipase C Assays

Hemolysis and phosphatidylinositol-specific phospholipase C (PI-PLC) activity assays were used as *in vitro* proxies for virulence. Hemolysis assays were performed using human red blood cells as previously described (Muchaamba et al., 2019). To compare PI-PLC activities, the strains were grown on oxoid chromogenic *Listeri*a agar plates. Overnight, BHI cultures of each strain were serially diluted in phosphate-buffered saline (10^8^ CFU/ml) and spotted (5 μl) on oxoid chromogenic *Listeri*a agar plates that were incubated at 37°C and visually examined after 48 h for the zone of opacity. The width of these zones was also measured. *L. monocytogenes* LL195 and *Listeria innocua* JF5051 strains were included as positive and negative controls, respectively. All hemolysis data were assessed relative to LL195. Experiments were conducted in triplicate on three separate occasions.

### Growth Evaluation Under Sodium Chloride, Lysozyme, and Benzalkonium Chloride Stress Conditions

Secondary stationary phase stage BHI cultures prepared as described earlier from each *L. monocytogenes* strain were diluted in BHI to 10^5^ CFU/ml. To assess growth under sodium chloride (NaCl), BC, or lysozyme stress, 100-μl volumes of BHI media supplemented with NaCl (0, 8, and 16%), lysozyme (40 μg/ml; Sigma-Aldrich), and BC (0–14 μg/ml) were added in triplicate to wells of a 96-well microplate to which 100 μl of the different *L. monocytogenes* strains at 10^5^ CFU/ml had been preadded. Cultures were incubated for 24 to 48 h at 37*^o^*C with shaking in a Synergy HT OD reader (BioTek Instruments, GmbH, Switzerland), and the OD_600_ was measured every 30 min. Dual NaCl and cold stress tolerance comparison was also made. A selection of strains was grown in BHI alone or in the presence of 4 and 8% NaCl using the 96-well plate setup and 10-ml tubes at 7°C for 505 h non-shaking. Growth was monitored through periodic OD_600_ measurement using the Synergy HT OD reader. To validate bacterial growth profiles observed in zebrafish embryos, the strains were also grown in BHI alone at 28°C. Growth kinetics such as lag phase duration, maximal growth rate, and area under the curve was determined from the OD_600_ growth data using the program GraphPad Prism and R (Göker, 2016; Göker et al., 2016).

Lysozyme sensitivity was also analyzed using disk diffusion assays on all the strains. One hundred microliters of a 10^8^ CFU/ml culture of each strain were plated onto BHI agar, and then, a sterile filter disk containing 1.25 mg of lysozyme was added. The plates were incubated for 24 h at 37°C, after which zones of inhibition were then measured. The diameter of the disk (6 mm) was considered as the zone for the resistant strains.

### Genome Analysis

Rapid Annotation Subsystem Technology (RAST) and Seed Viewer standard setting were used for genome annotation and comparisons. Genomes were aligned using CLC Genomics version 20.0.3. (Qiagen, Prismet, Denmark) whole-genome alignment tool using standard settings. Genes possibly linked to phenotypic differences were searched and compared between the genomes in CLC genomics and using BLASTn and BLASTp in the National Center for Biotechnology Information platform^1^. Coordinates for the positions of the SNPs, insertions, and deletions (InDels) were determined using this platform. Positions of amino acid (aa) changes on selected proteins were determined using the same approach. Where applicable, *L. monocytogenes* strains EGDe, LL195, and N2306 were used as the reference strains.

The pan-genome for 56 isolates (Supplementary Table 1) was generated using the *L. monocytogenes* BIGSdb platform^2^. Due to SNP-induced false discoveries of gene absence when using automated pipelines, the absence or presence of a gene was verified by manual blast using CLC genomics. The relatedness of the strains was further assessed through core genome MLST analysis, performed in accordance with the core genome previously defined (Ruppitsch et al., 2015). Sequences were blasted against 1,701 genes, and cluster types were determined in Seqsphere+ 7.7.5 (Ridom GmbH, Münster, Germany) (Ruppitsch et al., 2015) using standard settings. Missing genes were ignored in all samples. Minimum spanning trees were generated in SeqSphere+ for visualization of strain-relatedness. Clusters were defined as isolates containing ≤10 different alleles between a pair of isolates (Ruppitsch et al., 2015). An average nucleotide identity was calculated using CLC genomics (standard settings) and visualized as a circular phylogeny tree.

### Protein Structure Analysis

The three-dimensional (3D) protein structure of selected proteins known to be important for virulence and lysozyme and osmotic stress tolerance were analyzed using the Phyre2 web portal (Kelley et al., 2015). The effects of single aa substitutions on the structure of selected genes were predicted and modeled using the online server^3^ as previously described (Ittisoponpisan et al., 2019). The derived 3D protein structures were aligned and superimposed onto each other using an mTM-align web server and compared as previously described (Dong et al., 2018a,b). Template modeling scores (TM-scores) were used as a measure of similarity between the predicted protein structures. A TM-score of 1 signified a complete match between two structures. For the lineage-based protein structure comparison, *L. monocytogenes* strains LL195, EGDe, and LMNC318 were used to represent lineage I, II, and III strains, respectively.

### Statistical Analyses

All experiments presented were performed independently in triplicate at least three times unless stated otherwise. GraphPad Prism [version 9.2.0 (283), GraphPad Software, San Diego, CA, United States] was used for the statistical analysis of data. One-way analysis of variance with *post hoc* Tukey honestly significant difference tests was used to assess the statistical significance of differences between the strains. *P*-values < 0.05 were statistically significant. Data not normally distributed was normalized by log transformation before analysis.

## Results

### Study Strain Demographics

**FIGURE 1:**
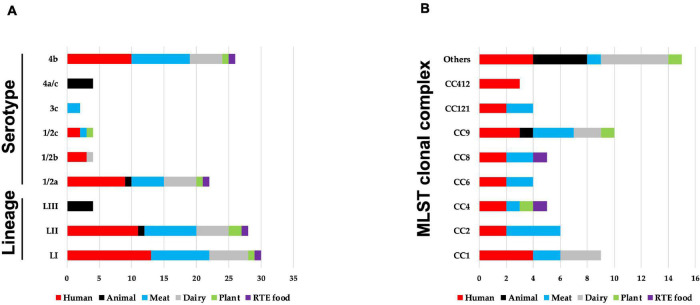
An overview of distribution of 62 *Listeria monocytogenes* strains examined in this study based on their **(A)** isolation source, genetic lineage, serotypes, and **(B)** MLST clonal complexes. **(B)** MLST clonal complexes with three or more strains are presented, whereas those with less are represented under others (CC3; CC5; CC7; CC18; CC20; CC29; CC70; CC131; CC217; CC226; and CC489).

Sixty-two strains selected to cover the genetic diversity of *L. monocytogenes* and representing both clinical and food-relevant clones were investigated (Supplementary Table 1). An overview showing the distribution of the examined strains based on isolation sources, serotypes, genetic lineage, and CCs is presented in Figure 1. The strains included human (*n* = 24) and animal (*n* = 5) clinical isolates and food isolates, from plants (*n* = 3), meat (*n* = 17), dairy (*n* = 11), and RTE (*n* = 2) food products. *L. monocytogenes* genetic lineages LI (*n* = 30), LII (*n* = 28), and LIII (*n* = 4), as well as 19 CCs, were represented among the strains.

### Virulence Variation Among *L. monocytogenes* Strains

#### *Listeria monocytogenes* Zebrafish Virulence Follows Genotype, Serotype, and Strain-Dependent Trends

**FIGURE 2:**
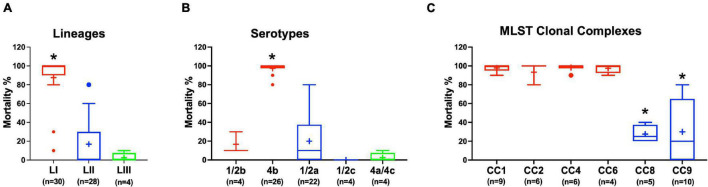
*Listeria monocytogenes* virulence in zebrafish varies with strain, serotype, and genotype. Presented are box plots showing virulence comparison based on **(A)** lineage, **(B)** serotype, and **(C)** clonal complex using zebrafish embryo infection model at 24 hpi. *Indicates that the group differs significantly from other groups (*P* < 0.05). Results are based on three independent biological repeats. Key; red: LI strains, blue: LII strains, green: LIII strains.

**FIGURE 3:**
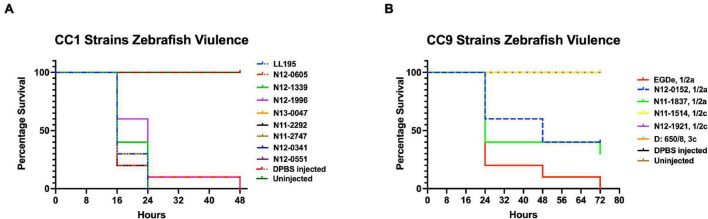
*Listeria monocytogenes* **(A)** CC1 and **(B)** CC9 strains display intra-clonal complex differences in zebrafish embryo virulence. Presented are Kaplan–Meier survival curves of zebrafish embryo (*n* = 10 per strain) infected (500 CFU) with different CC1 or CC9 *L. monocytogenes* strains and monitored for 3 days. Results are based on three independent biological repeats.

Virulence of the 62 *L. monocytogenes* strains was assessed using a zebrafish embryo-based infection model. LI strains were the most virulent, whereas LIII strains were the least virulent (Figure 2A). A mortality level trend of LI (85%) > LII (17%) > LIII (2.5%) was observed at 24 hpi (Figure 2A). At the serotype level, serotype 4b strains were more virulent than other serotypes (Figure 2B). All LI serotype 4b strains (*n* = 26) had achieved 100% mortality at 24–48 hpi. Even at 72 hpi, none of the examined LI serotype 1/2b (*n* = 4) and LIII strains (*n* = 4) had achieved 100% mortality, whereas only one of the examined LII strains (*n* = 28), *L. monocytogenes* EGDe, had caused 100% mortality (Supplementary Figure 1). Zebrafish virulence comparison of the strains clustered at MLST CC level also revealed both CC and strain-specific intra-clonal complex virulence variation. CC1, CC2, CC4, and CC6 strains displayed higher virulence, whereas CC8 and CC9 strains had lower virulence (Figure 2C and Supplementary Figure 1). Strain-dependent intra-clonal complex virulence differences were observed for strains in CC1 and CC9. Relative to the other CC1 strains, three CC1 strains (N11-2292, N12-0605, and N12-1996) were significantly impaired in virulence (Figure 3A). CC9, serotype 1/2a strains displayed varying virulence levels, whereas all six tested CC9 serotype 1/2c and 3c strains were avirulent (Figure 3B). Overall, our data show that *L. monocytogenes* virulence varies based on genotype, serotype, and strain.

#### *Listeria monocytogenes* Hemolytic Activity Trends Are Not Correlated With Zebrafish Virulence

**FIGURE 4:**
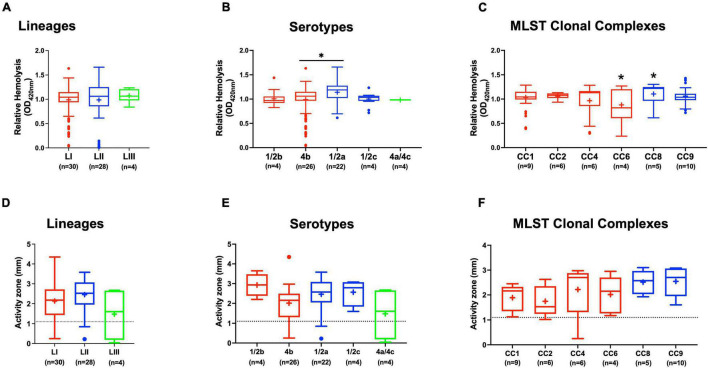
Hemolysis and PI-PLC activity comparison. Presented are box plots showing **(A–C)** relative hemolysis capacity and **(D–F)** PI-PLC activity comparison among strains. *Significant difference between groups (*P* < 0.05), based on one-way analysis of variance and Tukey *post hoc* test pairwise comparison. Results are based on three independent biological repeats. Key; red: LI strains, blue: LII strains, green: LIII strains. Dotted line denotes a low PI-PLC activity cutoff value.

Assessing hemolysis as a proxy for the virulence potential of the 62 strains showed that the *in vitro* determined hemolysis levels do not always correlate with the observed *in vivo* virulence ability of strains in the zebrafish infection model. Although serotype 1/2a and CC8 strains displayed significantly higher overall hemolytic capacity, they did not show correspondingly higher virulence than strains of other CCs upon infection of zebrafish embryos (Figures 4A–C). No significant differences were observed in hemolytic ability when the strains were grouped based on genetic lineages despite clear lineage-specific trends observed in zebrafish embryo virulence (Figures 2A, 4A). However, in agreement, non-hemolytic strains such as N05-195 and N843_15 were also avirulent in the zebrafish embryo infection assay. The strains were further compared with respect to PI-PLC activity revealing lineage and strain-specific variation. As a group, LIII strains displayed lower PI-PLC activity compared with LI and LII strains (Figures 4D–F). Notably, among these were LIII strains WLSC1019 and WLSC1020 that displayed the overall lowest PI-PLC activity (Supplementary Figure 2A). This might have contributed to both strains being avirulent in the zebrafish infection model. Strains N12-2747 (CC1), N12-0794 (CC4), and N12-1387 (CC6), despite demonstrating very low hemolytic and PI-PLC activity, were highly virulent in the zebrafish embryo-based assay (Supplementary Figure 2A).

#### Survival and Growth Trends of *L. monocytogenes* Strains Within Infected Zebrafish Embryos Correlate With Observed Virulence Capacity

**FIGURE 5:**
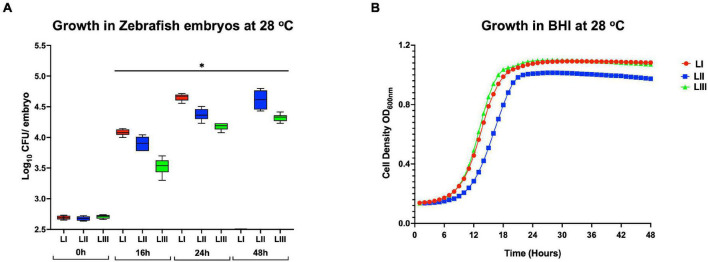
Growth and survival of *L. monocytogenes* strains from different genetic backgrounds within **(A)** zebrafish and **(B)** BHI at 28°C. Key; red: LI strains, blue: LII strains, green: LIII strains. *Significant difference between lineages and time points (*P* < 0.05). Results are based on three independent biological repeats.

**FIGURE 6:**
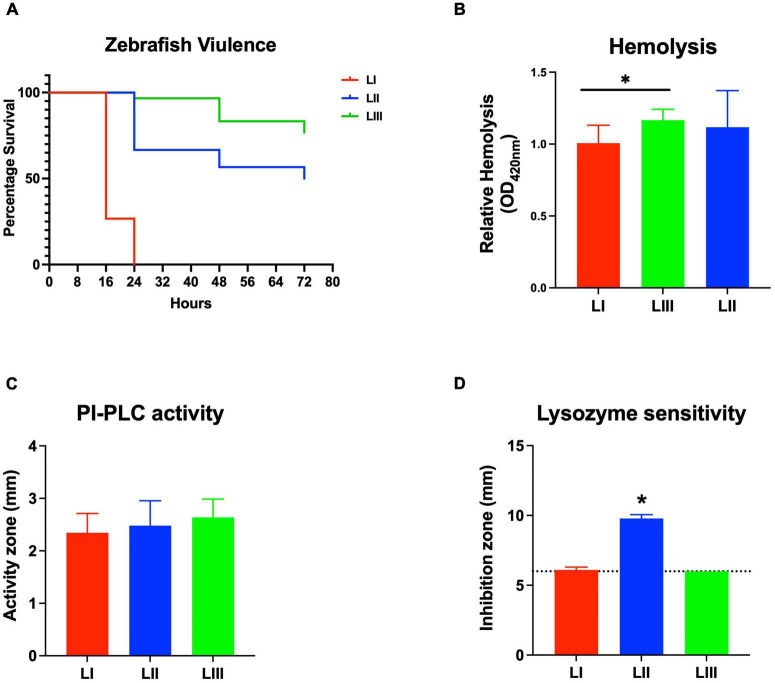
*Listeria monocytogenes* strains display lineage-based differences in zebrafish embryo virulence. Presented are **(A)** survival curves of zebrafish embryo (*n* = 10 per strain) infected (500 CFU) with different *L. monocytogenes* strains representing LI, LII, and LIII. Embryos were monitored for 3 days post-infection. **(B–D)** Hemolysis, PI-PLC activity, and lysozyme stress tolerance profiles of injected strains are also shown. **(D)** Dotted line denotes lysozyme resistant strain classification cutoff value. *Significant difference between **(B)** LI and LIII, **(D)** LII and the other lineages (*P* < 0.05). Results are based on three independent biological repeats.

Growth monitoring conducted with representative LI, LII, and LIII strains during the course of zebrafish infections revealed lineage-dependent survival and growth trends within infected zebrafish embryos. The observed trends mirrored zebrafish embryo mortality severity profiles induced by these strains. LI strains that induced the highest mortality (100% at 24 hpi) showed the highest survival and growth capacity, whereas LIII strains that induced the least mortality (5%) had the lowest survival and growth capacity within infected zebrafish embryos (Figures 5A, 6A). Growth and survival ability within the zebrafish embryos for the strains ranked in the order LI > LII > LIII. Growth kinetics determined in BHI broth cultures with these LI, LII, and LIII representative strains at 28°C, the incubation temperature applied in the zebrafish infection model, showed that there was no correlation between virulence and growth capacity in zebrafish embryos with strain growth behavior in BHI at 28°C (Figure 5B). As such, virulence as well as survival and growth behavior differences observed between *L. monocytogenes* strains in the zebrafish infection model were not attributed to bacterial growth capacity differences due to the applied experimental incubation temperature conditions. A further comparison of these strains used for tracking bacterial growth within the zebrafish embryos also showed that their hemolysis and PI-PLC activity were not correlated with growth and survival capacity within the embryos (Figures 6B,C).

### Stress Resilience Variation Among *L. monocytogenes* Strains

#### Lysozyme Stress Sensitivity Variation

**FIGURE 7:**
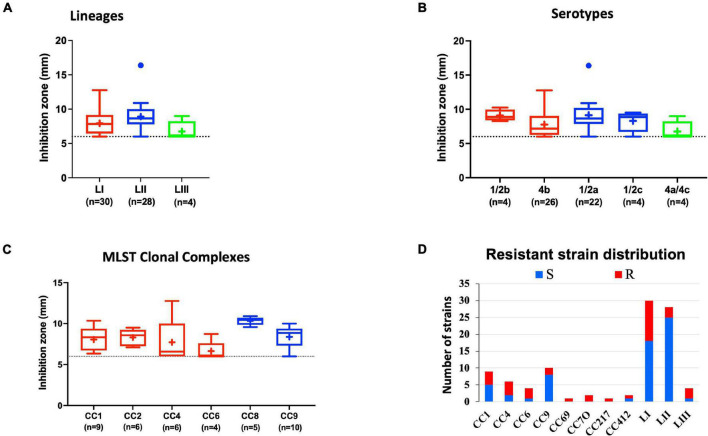
Lysozyme stress sensitivity of *L. monocytogenes* varies with strain and molecular subtype. Presented are box plots showing lysozyme stress sensitivity comparison based on **(A)** lineage, **(B)** serotype, and **(C)** clonal complex. **(D)** Shows distribution of strains resistant to lysozyme. Key; red: LI strains, blue: LII strains, green: LIII strains. Results are based on three independent biological repeats.

Comparing lysozyme stress sensitivity among the 62 *L. monocytogenes* strains, besides uncovering individual strain-dependent variation, also showed genetic lineage and CC-associated trends (Figure 7). More LI strains (12/30) and LIII (3/4) were resistant to lysozyme stress compared with LII strains (Figure 7D). Only three LII strains (*n* = 28) were resistant to lysozyme. CC8, CC9, and CC121 strains showed increased sensitivity to lysozyme compared with the CC1, CC4, and CC6 strains. Strain-dependent variations in lysozyme stress tolerance were detected within the same serotype, genetic lineage, and CC groups. For example, among all tested strains, N84_10 was the most sensitive, whereas its close relative isolate N843_15 was resistant to lysozyme stress (Supplementary Figure 2B). CC9 serotype 1/2a strains were more sensitive to lysozyme than CC9 serotype 1/2c strains (Supplementary Figure 3). Of the strains used to track bacterial growth within the zebrafish, LII strains were more sensitive to lysozyme compared with LI and LIII strains (Figure 6D). Surprisingly, tolerance trends generated from the growth of the 62 strains in BHI under low levels of lysozyme stress were in most parts inversely correlated to the disk diffusion trends (Supplementary Figure 3). However, they corroborated that the study strains have lineage, serotype, CC, and strain-based differences in sensitivity to lysozyme stress.

#### Variation in NaCl Stress Sensitivity

**FIGURE 8:**
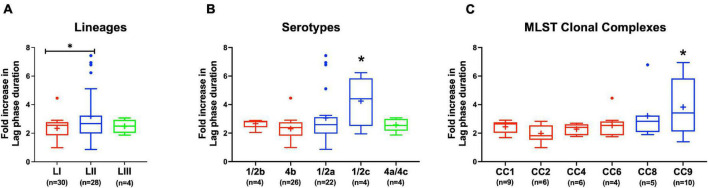
*Listeria monocytogenes* osmotic stress tolerance varies with strain and molecular subtype. Presented are box plots showing comparison of fold increase in lag phase duration of strains due to osmotic stress (growth in BHI plus 8% NaCl), **(A)** lineages, **(B)** serotypes, and **(C)** clonal complexes. *Significant difference between **(A)** LI and LII, **(B)** serotype 1/2c and other serotypes and **(C)** CC9 and other CCs (*P* < 0.05), based on one-way analysis of variance and Tukey *post hoc* test pairwise comparison. Results are based on three independent biological repeats. Key; red: LI strains, blue: LII strains, green: LIII strains.

**FIGURE 9:**
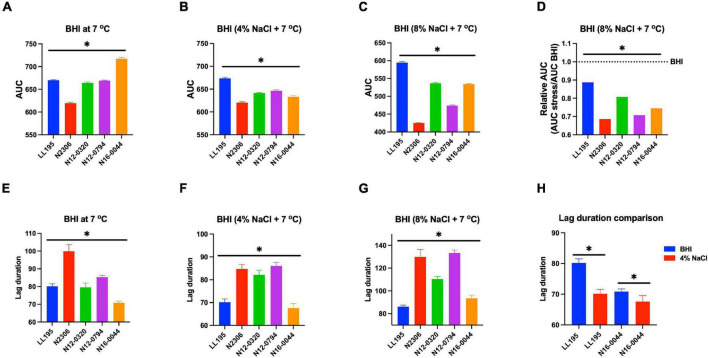
Comparison of serotype 4b *L. monocytogenes* strains [N2306, N12-0320, and N12-0794 (CC4), LL195 (CC1), and N16-0044 (CC6)] growth profiles at low temperature with or without NaCl osmotic stress based on **(A–D)** area under curve and **(E–H)** lag phase duration extension. *Significant difference between strains and treatments (*P* < 0.05). Results are based on three independent biological repeats.

Evaluating NaCl salt stress (BHI plus 8% NaCl), sensitivity in BHI cultures at 37°C showed strain, serotype, and genotype-dependent salt stress tolerance variation across the 62 *L. monocytogenes* strains. Comparing NaCl stress-induced increase in lag phase duration and area under the curve reduction during growth in BHI, LI, serotype 4b, CC2, and CC4 strains were most tolerant, whereas LII, serotype 1/2c as well as CC9 and CC121 strains were most sensitive to NaCl stress (Figure 8 and Supplementary Figure 4). The observed NaCl stress tolerance trends interestingly mirrored virulence patterns seen in the zebrafish embryo infection model (Figures 2, 8). The more osmotolerant LI serotype 4b strains were also more virulent than other lineage and serotype strains. On the other hand, the least osmotolerant serotype 1/2c and CC9 strains were also the least virulent strain group (Figures 2, 8). Strain-associated variation trends in NaCl stress tolerance were similarly observed during growth under NaCl stress at low temperature (7°C) related to food preservation (Figure 9). LL195, a previous listeriosis outbreak strain, was notably more tolerant to cold and salt stress combination compared with other tested strains (Figure 9). Moreover, this strain and other strains such as N16-0044 exhibited shortened lag phase (LL195; 70 vs. 80 h) under salt stress compared with growth in BHI without salt stress during incubation at low temperature (Figures 9E–H). An observation consistent with enhanced adaptation and growth of *L. monocytogenes* cells at low temperature when exposed to low salt concentration associated osmotic stress.

#### Strain-Dependent Variation in Benzalkonium Chloride Stress Tolerance

**FIGURE 10:**
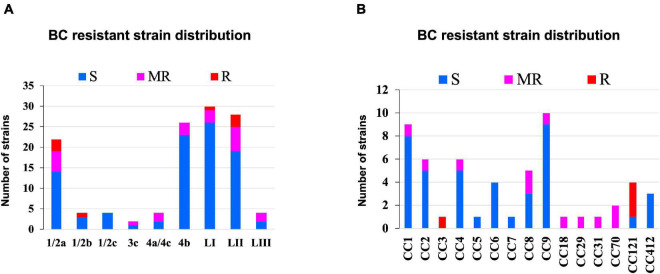
Distribution of strains resistant to BC stress based on **(A)** lineage and serotype **(B)** clonal complex.

Benzalkonium chloride is commonly used as a sanitizer in a food production environment; resistance toward it was assessed based on minimum inhibitory concentrations (MIC) determined in BHI broth. Strains displaying MICs below 4 μg/ml BC were considered sensitive, whereas those that grew at or above this concentration were considered BC resistant. Based on this classification, 11/62 strains were moderately resistant (MIC = 4 μg/ml), whereas 4/62 (MIC = >4 μg/ml) were considered highly resistant (Figure 10 and Supplementary Table 1). Eight of these moderate to highly resistant strains (*n* = 15) were from meat and milk samples or their products, whereas seven of these isolates were from clinical cases. Therefore, strain source had no influence on BC sensitivity. Notably, two listeriosis outbreak strains (N1546 and Lm3136) were among the BC-resistant strains. LI, serotype 1/2b strain N14-0435 (CC3), and three of the four tested LII CC121 strains (N11-1905, N13-0119, and N12-0367) displayed the highest tolerance to BC stress (Figure 10 and Supplementary Table 1).

### Linking Phenotypic Variation to Genomic Differences Among the Study Strains

#### Differences in Virulence Determinants

**FIGURE 11:**
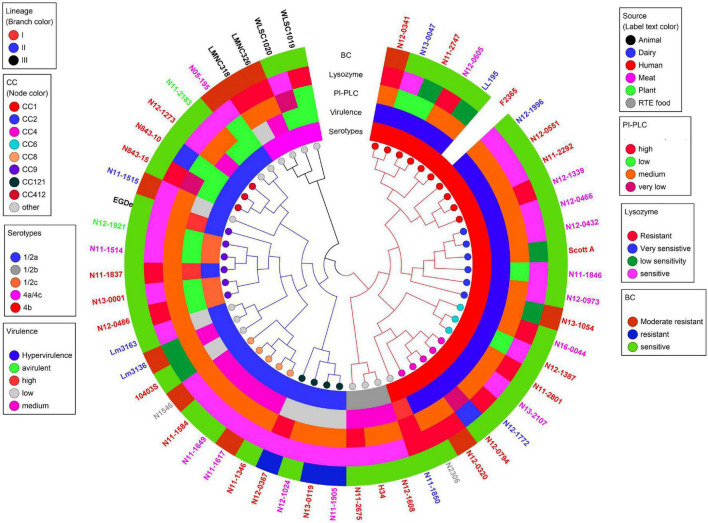
Cladogram showing genetic relatedness among *L. monocytogenes* strains examined in this study based on average nucleotide identity. Strain distribution with respect to lineage (branch line color), CC (node color), serotypes, isolation sources (label text color), zebrafish virulence, PI-PLC activity, lysozyme, and BC resistance is shown.

*Listeria monocytogenes* strains were subjected to genome analysis to examine for genetic variation associated with the observed virulence and stress resistance phenotypic differences. Strain relatedness visualized through core genome MLST and average nucleotide identity-based phylogenetic trees grouped the strains according to genetic lineages, serotypes, and CCs (Figure 11 and Supplementary Figure 5). Gene presence and absence patterns comparison of major virulence and stress tolerance-associated genes showed high conservation between strains, suggesting that the observed phenotypic variation was probably due to minor genetic and metabolic pathway variation (Supplementary Tables 2, 3). Nonetheless, gene content comparison at this level showed strain and genetic background-specific gene content differences involving some important virulence and stress tolerance-associated genes (Table 1 and Supplementary Tables 2, 3). Although all strains possessed the LIPI-1 island, some strains had truncated *prfA* (LII; N05-195, N843_10, and N843_15) or *plcB* (LI; H34) genes (Supplementary Tables 4–9). LIPI-3 was exclusive to LI strains, except for the examined CC2 strains and a CC5 1/2b strain (N11-2675). The LIPI-3 genes in some strains, however, encoded truncated proteins. A CC1 strain N11-2747 has a truncated IIsP at aa position 102 (length: 101aa vs. 183aa in LL195), whereas N13-2107 a CC4 strain, has a truncated IIsX at aa position 18 (length: 17aa vs. 105aa in LL195). It is possible that such mutations might contribute to reduced virulence. Strain N11-2747, for example, had an overall 20% lower mortality induction compared with the most virulent CC1 strain at 16 hpi in zebrafish embryos (Figure 3A). LIPI-4 encoding cellobiose-type phosphotransferase systems postulated to enhance invasion, leading to neural and placental listeriosis, occurred only in CC4 and CC217 strains. Variable combination and content of internalin genes was observed due to the presence or absence of *inlC*, *inlC*2, *inlD*, *inlE*, *inlF, inlG*, *inlH*, *inlJ*, *inlI*, *inlP*, and *inlL* (Supplementary Tables 2, 10, 11). Except for three CC6 (N11-2801, N12-1387, and N16-0044) strains, one of which (N16-0044) had a truncated *inlG* gene, this gene was absent from LI strains. In contrast, all LI and LIII strains as well as LII strains Lm3136, N05-195, CC8, and CC121 strains lacked *inlL* (Supplementary Table 2). CC121 strains also lacked *inlF* and *inlG*.

**TABLE 1:** Strain and lineage genomic differences linked to phenotypic variability^a^.

Gene or Island	Lineage I	Lineage II	Lineage III
LIPI-1^b^	(1) 27/28	(3) 21/24	4/4
LIPI-3^c^	(2) 20/28	0/24	0/4
LIPI-4	6/28	0/24	0/4
*inlA*	28/28	14/24	4/4
*inlB*	27/28	24/24	4/4
SSI-1	3/28	15/24	3/4
*cspA*, *cspB*, *cspD*	28/28	24/24	4/4
BC resistance genes	1/28	4/24	0/4
*lmo1080-1084* cassette^d^	3/28	(2) 24/24	0/4

*^a^Only a few selected genomic differences are presented. For more details, refer to Supplementary Tables 2, 3.*

*^b^(1) 27/28 means 27 of 28 LI strains have a LIPI-1 with all full-length proteins, whereas 1 strain has at least one truncated LIPI-1 protein. LI strain H34 encodes a truncated PlcB, whereas LII strains (N843_10, N843_15, and N05-195) encode a truncated PrfA.*

*^c^Two of 20 strains encoded truncated proteins, strain N11-2747 has a truncated IisP, whereas N13-2107, a CC4 strain, has a truncated IisX.*

*^d^Two of 24 strains encoded truncated proteins, strain N843_10 and N843_15 encode truncated RmlB (Lmo1083) proteins.*

Strain and genetic background-associated SNPs were detected in virulence-associated genes, including the LIPI-1 as well as *inlA* and *inlB* genes. Some of these genetic variations might be functionally relevant and contribute to some of the virulence phenotypic differences observed between strains (Table 2 and Supplementary Tables 4–11). Examples include lineage and clonal complex-specific SNP-induced aa substitutions in LLO such as the L35S and I438V detected only in LI strains and the V433I substitution that was unique to the examined CC412 strains (Supplementary Table 5). An R3K aa substitution in InlA was only detected in LI strains, whereas Y774D was observed in CC4 and CC6 strains. In addition, all CC6 strains had a triple aa deletion in the pre-anchor region of InlA, the impact of which is yet to be established (Supplementary Table 10). Compared with the *L. monocytogenes* reference strain EGDe, 10 strains had altered InlA proteins. Strain N05-195 (CC31 serotype 1/2a) had a 612aa C-terminal InlA truncation (length: 188aa vs. 800aa) due to a PMSC, whereas strain N11-1921 (CC9 serotype 1/2c) had a 684aa long InlA also due to a PMSC. A PMSC truncating InlA at 576aa was detected in four other CC9 serotype 1/2c strains (N12-0486, N11-1837, N13-0001, and N11-1514). All CC121 strains (N11-1905, N12-1024, N13-0119, and N12-0367) encoded a truncated InlA 491aa long. Comparing these to known *inlA* PMSC mutation types (Gelbíčová et al., 2015) identified mutation types 5 (188aa), 6 (491aa), 11 (684aa), and 12 (576aa). All the 10 strains were avirulent or showed low virulence (CC121 strains) in the zebrafish embryo infection model (Figure 11). Due to a PMSC, strain N12-1608 encoded a truncated InlB (396aa vs. 630aa), which could have contributed to its reduced virulence relative to the hypervirulent LI strains.

**TABLE 2:** Genetic background-associated SNPs or InDels in genes important for *Listeria monocytogenes* virulence.

Virulence factor^a^	Encoding gene^b^	Function/annotation	Number of genetic background-based SNPs or InDels^c^
PrfA	*lmo0200*	Virulence gene expression regulator	1
LLO	*lmo0202*	Listeriolysin O	4
PlcA	*lmo0201*	Phosphadidylinositol phosphodiesterase (PI-PLC)	3
PlcB	*lmo0205*	Phosphatidylcholine phospholipase C (PC-PLC)	13
Mpl	*lmo0203*	Zinc-metalloprotease	24
ActA	*lmo0204*	Actin-based motility and cell-to-cell spread	48
InlA	*lmo0433*	Internalin A: cell invasion	16
InlB	*lmo0434*	Internalin B: cell invasion	41
PdgA	*lmo0415*	Deacetylates: cell wall modification	58
OatA	*lmo1291*	Peptidoglycan *O*-acetyltransferase	48
Hpt	*lmo0838*	Hexose phosphate transport protein	4
MdrM	*lmo1617*	Multidrug resistance transporter	1
LapB	*lmo1666*	*Listeria* adhesion protein B	64
Iap^d^	*lmo0582*	Invasion associated protein	30
LisRK	*lmo1377-lmo1378*	Two component regulatory system	1^e^
VirRS	*Lmo1745, lmo1741*	Two component regulatory system	6
DltABCD	*lmo0971-lmo0974*	Incorporation of D-Alanine in wall teichoic acids	80
OppA^d^	*lmo2196*	Oligopeptide transporter	2
Ctc^d^	*lmo0211*	General stress protein, L25 family of ribosomal protein	Strain specific
**Sec system**			
SecY	*lmo2612*	Preprotein translocase subunit SecY	2
SecE	*lmo0245*	Preprotein translocase subunit SecE	1
SecG	*lmo2451*	Preprotein translocase subunit SecG	Conserved
SecDF	*lmo1527*	Preprotein translocase SecDF	12
YajC	*lmo1529*	Preprotein translocase subunit YajC	7
YidC	*lmo1379*	YidC/Oxa1 family membrane protein insertase	1
SecA	*lmo2510*	Preprotein translocase subunit SecA	3
SecA2	*lmo0583*	Protein-secreting ATPase	16
PrsA2	*lmo2219*	Secreted bacterial lipoprotein chaperone	7
Lsp	*lmo1844*	Lipoprotein signal peptidase	3
LspB	*lmo1101*	Lipoprotein signal peptidase	Detected in EGDe only

*^a^A selection of L. monocytogenes virulence factors is presented.*

*^b^Based on L. monocytogenes reference strain EGDe.*

*^c^Differences reported are SNPs resulting in amino acid changes in encoded protein enriched in a particular lineage/s or clonal complex/es.*

*^d^Also important for osmotic stress tolerance. For more details on SNPs and Indels annotations, see Supplementary Tables 4–33.*

*^e^lisR gene has no genetic background-based differences.*

Genotype (lineage and CC) and strain-specific genetic differences were also detected in several other virulence factor genes, including *pgdA*, *oatA*, *hpt*, *inlC*, *tagB*, *iap*, *lapB*, *aut*, *ami*, and *lisRK* (Table 2 and Supplementary Tables 2, 12–18). Although functional consequences for most of these changes are not yet known, they might also contribute to the strain and genotype-specific virulence differences observed. The *ami* gene involved in cell adherence was present in all strains; however, it had several genetic background-associated InDels leading to aa length variations. In LI strains, it was 770aa long except for N11-1846 (CC2; 605aa) and the serotype 1/2b strains N12-1608, H34, and N11-2675 (917aa). In LIII, *ami* encoded a 935aa long protein, whereas in LII, it was 917aa except for N05-195 (CC31), N11-1346 (CC8), and CC121 strains (N12-0367 and N13-0119), which had a 596aa long Ami. Serotype 4b strains contained *aut IVb*, an allele of the invasion gene *aut*, but lacked teichoic acid biosynthesis-associated gene *tagB*. LI serotype 1/2b and LIII strains all lacked serotype 4b-specific teichoic acid biosynthesis genes *gltA* and *gltB.* The examined CC31 (N05-195), CC412 (N12-1273, N843_10, N843_15), and LIII (LMNC318, LMNC326, WLSC1019, and WLSC1020) strains all lacked the adherence gene *lapB*. In contrast, the gene in strain Lm3163 (CC18) encodes a truncated LapB protein (841aa vs. 1711aa) due to a PMSC, whereas in strain N13-0047 (CC1), it encoded a shortened protein (1703 vs. 1711aa) due to a 27 nucleotide in-frame deletion at position 3,246–3,272, which resulted in a 9aa deletion. The size of LapB in LII was conserved (1711aa), whereas LI strains carry LapB of varying size (1,712–1,718aa) due to several InDels in the nucleotide region 4,922–4,963 of their *lapB* genes (Supplementary Table 16). Significant InDel-induced protein size and sequence variations were also detected among the study strains in other virulence factors such as the invasion-associated protein Iap (454–484aa) and ActA (604–639aa) (Supplementary Table 17). The VirRS and DltABCD system, by altering the cell surface charge, can aid in the evasion of the host immune system. Genetic background-associated SNPs were also detected in genes encoding for these systems (Table 2 and Supplementary Tables 19–24). Overall, LIII strains had the fewest number of virulence factors (WLSC1019: 42, WLSC1020: 51, LMNC318: 53, and LMNC326: 53), corresponding to them showing the lowest virulence in the zebrafish embryo infection model (Figure 2 and Supplementary Table 2). Apart from LIPI-3 and LIPI-4, these strains also lacked *tagB*, *aut*, and *inlF* genes. Meanwhile, LIII strains WLSC1019 and WLSC1020 as well as N05-195 (LII, CC31) that were all avirulent in the zebrafish infection model also encoded truncated versions of InlC. LI, CC1 strains N12-1996 and N12-0432, LII strain 10403S and LIII strains WLSC1019 and WLSC1020 encode truncated ArgA proteins (13aa, 94aa, 214aa, 214aa, and 124aa long, respectively). N12-1996 a LI, CC1 serotype 4b strain, and WLSC1019 (LII) encoded a truncated or lacked the *inlJ* gene, respectively.

Protein secretory systems play an important role in the delivery of virulence factors to their site of action. Excluding *lmo1101* that encodes LspB, a signal peptidase which was only detected in *L. monocytogenes* EGDe, full-length proteins that make up these systems were detected in all examined strains (Supplementary Table 2). However, aa altering sequence differences were observed in these genes. For instance, several strain and genetic background-associated SNPs and InDels were detected in genes encoding Sec system components and chaperons such as SecA2, IspA, YajC, YidC, LspA, and PrsA2 (Table 2 and Supplementary Tables 2, 25–33). Restriction modification systems (RMS) and DNA methylation enzymes can alter gene expression through epigenetic modifications. Clone-specific RMS and methylation enzymes were detected among the strain. For example, CC1, CC2 as well as CC4 and CC8 carry such clone-specific RMS. DNA adenine methylase encoding genes *lmo1119* and *lmo2316* were only detected in CC9 strains and *L. monocytogenes* EGDe strain, respectively (Supplementary Table 2).

#### Genetic Variation in Stress Tolerance Determinants

OatA and PgdA proteins are important cell wall modification enzymes involved in antimicrobial peptide and lysozyme tolerance. The *pgdA* and *oatA* genes among the examined strains had lineage-associated aa altering SNPs, which might alter the activity of these enzymes in a lineage-based manner (Supplementary Tables 12, 13). Strain N12-0431 encoded a truncated OatA protein (210aa vs. 622aa) but did not show increased lysozyme sensitivity, probably because it still possesses an intact PgdA. WTA glycosylation also promotes *L. monocytogenes* resistance against host and food-associated antimicrobial peptides aiding in evasion of the host immune system. Genes encoding proteins involved in these protective processes, such as *gtcA* and the *rmlACBD* locus and *rmlT* (*lmo1080*–*1084*), showed genetic background-associated SNPs or distribution among the study strains (Table 1 and Supplementary Table 2). The *rmlACBD* locus and *rmlT*, which encodes the biosynthetic pathway for L-rhamnose and a putative rhamnosyltransferase, respectively, only occurred in LII and LI serotype 1/2b strains. The *gtcA* gene encoding the wall teichoic acid glycosylation protein GtcA, although present in all strains, had genetic background-specific SNPs. Such differences, among other effects, might contribute to survival and growth capacity differences observed between strains within infected zebrafish embryos. Possible phenotypic links to such genetic variations, however, remain to be investigated.

Strain and genetic background-associated SNPs were also identified in genes encoding for *L. monocytogenes* osmotic stress adaptation proteins or systems. These might also have functional consequences contributing to some of the NaCl stress tolerance phenotypic variation observed (Table 3 and Supplementary Tables 34–65). Genes for the general stress response regulator SigB (σ*^B^*) encoded truncated proteins in strains N2306, N11-1515, and N11-2747, which might contribute to increased general stress sensitivity. Strain N2306 (LI CC4 serotype 4b), for example, showed increased osmotic and cold stress sensitivity compared with the other tested LI strains (Figure 9). SSI-1 includes genes that promote *L. monocytogenes* growth under suboptimal host and food-related stress conditions. Lacking from LI serotype 4b strains, SSI-1 occurred in all LII and LIII as well as LI serotype 1/2b strains. An exception to this trend was the LII CC20, CC29, CC121, CC412, and LIII CC69 strains that lacked SSI-1 (Supplementary Table 3). Genes for BetL, OpuC, Gbu, ProBA, and TreA and the oligopeptide transporters OppA and DtpT that aid in biosynthesis and accumulation of osmoprotective molecules, such as betaine, carnitine, proline, and trehalose, also carried various strain and genetic background-associated sequence differences (Table 3 and Supplementary Tables 35–53). Strain N12-0341 (CC1) has a T insertion between nucleotide positions 1,419 and 1,420 of *dtpT* (*lmo0555*) that disrupts the regular protein stop codon resulted in a 25aa longer DtpT in N12-0341 compared with all other strains (Supplementary Table 50). Similarly, TreA protein in N05-195 (CC31) and analyzed CC412 strains were elongated by 4aa (Supplementary Table 52). Strain N12-1387 poses a truncated OppA protein (291aa vs. 588aa) due to an A insertion between nucleotide positions 848 and 849 of *oppA* (*lmo2196*) gene that gives rise to a PMSC (Supplementary Table 53). Strain and genetic background-associated aa differences were detected in the transcriptional regulator Lmo0501, also involved in osmotic stress responses, with strain N12-1921 encoding a truncated protein (Supplementary Table 54). Several genetic background-associated SNP-induced aa changes were also detected in Lmo1078 and Lmo2085, proteins involved in cell wall remodeling in response to osmotic stress (Table 3 and Supplementary Tables 55, 56). For example, the gene for Lmo1078 encoded an elongated protein (295aa vs. 290aa) in LI and LIII strains compared with LII (Supplementary Table 55). Both the Kdp system involved in rapid potassium ion (K^+^) importation, as well as the MrpABCDEFG sodium/proton antiporter, also play important roles in *L. monocytogenes* osmotic stress adaptation. Genetic background-associated sequence differences that may have functional consequences were also detected in genes that encode protein components of these two systems (Table 3 and Supplementary Tables 57–65). Genes for the efflux pump MdrM also had strain and lineage-associated aa changing SNPs that might also have yet unknown functional consequences in stress resilience and virulence phenotypes (Table 2 and Supplementary Table 15). Only 5 (N13-1054, N05-195, N11-1905, N13-0119, and N12-0367) of 15 BC-resistant strains carried a known classical BC resistance-conferring gene, that is, *Tn*6188_*qac* (*ermC*) (Table 1 and Supplementary Table 3). Other BC-tolerant strains, including the two listeriosis outbreak strains N1546 and Lm3136, did not carry any of the known BC resistance-conferring genes.

**TABLE 3:** Genetic background-associated SNPs or InDels in *L. monocytogenes* osmotic stress response systems encoding genes.

Stress response system^a^	Encoding gene^b^	Function/annotation^a^	Number of genetic background- based SNPs or InDels^c^
BetL	*lmo2092*	Glycine betaine transporter	10
Gbu	*lmo1014* – *lmo1016*	Glycine betaine transporter	5
OpuC	*lmo1425* – *lmo1428*	Carnitine transporter	4
KdpED	*lmo2678* – *lmo2679*	Kdp ATPase, two-component system for K+ uptake	36
KdpABC	*lmo2680* – *lmo2682*	Potassium-transporting ATPase	21
ProBA	*lmo1259* – *lmo1260*	Proline biosynthesis	31
OppA	*lmo2196*	Oligopeptide transporter	2
DtpT	*lmo0555*	Oligopeptide transporter	14
TreA	*lmo1254*	Phosphotrehalase	17
Lmo0501	*lmo0501*	Transcriptional regulator	4
Lmo1078	*lmo1078*	UDP-glucose phosphorylase	65
Lmo2085	*lmo2085*	LPXTG peptidoglycan bound protein	55
HtrA	*lmo0292*	General stress response serine protease	2
ClpC	*lmo0232*	General stress response protein ATPase	6
ClpP	*lmo2468*	General stress response protease	1
CspA	*lmo1364*	Cold shock protein	Conserved
CspB	*lmo2016*	Cold shock protein	Strain specific^d^
CspD	*lmo1879*	Cold shock protein	Conserved
RelA	*lmo1523*	(p)ppGpp synthetase	4
Ctc	*lmo0211*	General stress protein, L25 family of ribosomal protein	Strain specific
LisRK	*lmo1377* – *lmo1378*	Two component regulatory system	1^e^*
Iap	*lmo0582*	Invasion associated protein	30
MrpABCDEFG	*lmo2378* – *lmo2384*	Na^+^/H^+^ antiporter	15*
σ^B^	*lmo0895*	Alternative sigma factor σ^B^	1

*^a^Inclusion and function/annotation based on review by Burgess et al. (2016).*

*^b^Based on L. monocytogenes reference strain EGDe.*

*^c^Differences reported are SNPs resulting in amino acid changes in encoded protein enriched in a particular lineage/s or clonal complex/es.*

*^d^Only L. monocytogenes N11-2675 and N12-1608, contained an amino acid changing SNP in CspB.*

*^e^LisRK is also important for virulence. For more details on SNPs and Indels annotations, see Supplementary Tables 34–65.*

*All other genes in these operons have genetic background-based differences except for LisR, MprB, and MprF, conserved in all analyzed strains.

### Predicted Protein Structural Changes

**FIGURE 12:**
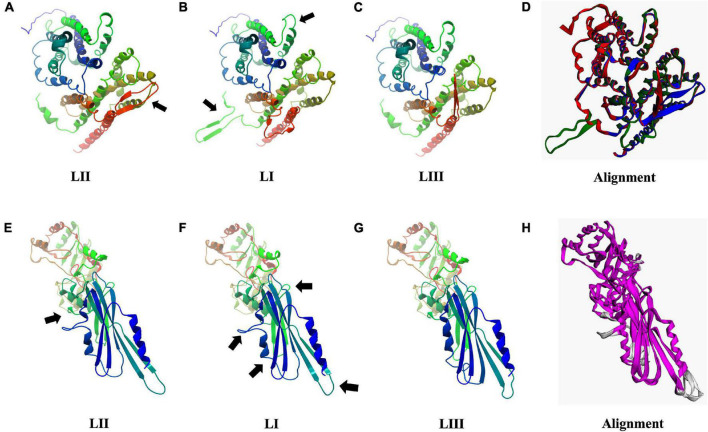
Consequences of aa changing SNPs on 3D protein structure of Hpt **(A–D)** and PgdA **(E–H)**. Black arrows highlight areas of most significant change. **(D,H)** 3D protein structure alignment. Key; for Hpt alignment **(D)** LI (green), LII (blue), and LIII (red). For PgdA alignment, **(H)** conserved chain regions are colored magenta, TM-score LI (0.9756), and LIII (0.9689) relative to LII.

As discussed earlier, SNP and InDel-induced aa changes resulted in strain and genetic background-associated structural changes in important virulence and stress tolerance proteins (Figure 12 and Supplementary Figures 6–13). Predicted 3D Iap protein structures showed significant structural differences between the LIII strains LMNC318 and WLSC1019 proteins (Supplementary Figure 8). At the lineage level, significant differences were also determined for predicted Hpt, PgdA, Iap, BetL, Lmo2085, Lmo1078, and PlcB protein structures. For instance, PlcB showed significant lineage-based structural differences, TM-scores LI (0.9431) and LIII (0.7627) relative to LII (Supplementary Figure 11). It is plausible that some of these predicted protein structural changes will have functional consequences contributing to the observed variation in virulence and stress tolerance phenotypes. Such a hypothesis would, however, have to be investigated through further experimental work. Hpt, a virulence factor important for intracellular growth by facilitating sugar–phosphate transportation, was predicted to have significant lineage-based structural changes, TM-scores of 0.9726 (LI) and 0.9840 (LIII) relative to LII (Figures 12A–D). Similarly, PgdA, important for lysozyme stress, also had such genetic background-based aa changes (Figures 12E–H). These changes might potentially alter Hpt and PgdA functions in a lineage-based manner contributing to host growth profile differences observed between strains within infected zebrafish embryos. On the other hand, the predicted 3D protein structures of other important proteins such as σ*^B^* (TM-score 0.9982), PrfA, LLO, and PlcA were highly conserved (TM-score 1; Supplementary Figure 13).

Csps are global regulatory proteins deployed to facilitate stress and virulence responses in *L. monocytogenes*. Csps were highly conserved with the exception of two LI serotype 1/2b strains N11-2675 and N12-1608 that had an SNP-induced single aa change (G22S) in CspB. Three-dimensional protein structure prediction and comparison with CspB of the reference strain EGDe showed that the G22S change did not significantly affect the structure of CspB in strains N11-2675 and N12-1608 (TM-score 1) and is therefore not expected to alter its functions.

## Discussion

To decipher the reason for variable distribution and virulence potential of *L. monocytogenes* subtypes, 62 strains recovered from food and clinical cases were characterized based on stress resistance and virulence phenotypes and through comparative genome analysis. LI serotype 4b strains commonly implicated in outbreaks and clinical human listeriosis cases showed the highest pathogenicity compared with others in the applied zebrafish embryo infection model. Meanwhile, the LII strains from CC8, CC9, and CC121 were the least virulent among examined strains in this infection model. Such genetic background-associated *L. monocytogenes* virulence trends are consistent with previous reports from studies that used mouse and *G. mellonella*-based virulence models (Maury et al., 2016; Lee et al., 2019). Notably, such virulence trends are also reflective of the epidemiological distribution of *L. monocytogenes* subtypes among human illness where the more virulent serotype 4b strains are also more enriched in clinical isolates (Maury et al., 2016).

Various strain and genotype-associated differences in nutrient utilization, virulence, and stress tolerance genes were uncovered through genome analysis. For instance, extra pathogenicity islands (LIPI-3 and LIPI-4) detected only in LI strains might be related to their increased virulence. Survival and growth differences within an infected host, as well as other genetic variations and expression differences among key virulence factors between strains, might also contribute to observed virulence variation between strains examined in this study. Comparing *in vitro* determined hemolytic capacities between strains showed that this virulence proxy was not predictive of observed *in vivo* virulence levels in the applied zebrafish embryo infection model. LLO production capacity differences were, therefore, not the main explanation for observed virulence phenotypic differences in this infection model. In fact, the hypervirulent LI strains of our study showed significantly lower hemolysis activity than LII serotype 1/2a, CC8 strains that showed low virulence. Interestingly, *L. monocytogenes* strains carrying mutations that result in over production and/or increased cytosolic activity of LLO induce necrosis faster and are significantly attenuated in virulence (Glomski et al., 2002, 2003; Schnupf et al., 2006; Bécavin et al., 2014). Moreover, these observations support the notion that *in vitro* assays are not sufficient for evaluating or predicting the pathogenic potential of *L. monocytogenes* strains (Liu et al., 2007; Bécavin et al., 2014).

On the other hand, growth capability differences within infected zebrafish embryos mirrored virulence capacity differences observed between *L. monocytogenes*’ genetic lineages. The most virulent LI strains also grew significantly more than LII and LIII strains in infected zebrafish embryos. Genetic background-associated differences in growth profiles within other hosts were also previously observed. There have been suggestions that some hypervirulent serovar 4b strains might have prolonged survival capacity within infected mice, thereby increasing secondary dissemination likelihood leading to more severe negative health outcomes (Vázquez-Boland et al., 2020). Survival and growth potential differences within the host might be linked to LI strains being better at evading the host immune system leading to reduced pathogen-associated molecular patterns production. Lineages might differ in cell wall modification abilities that prevent shedding or secretion of immunostimulatory peptides (McDougal and Sauer, 2018). Induced host cell death through apoptosis, autophagy, or pyroptosis might release cytokines attracting immune cells to the infection site as well as eliminate the replication niche for a pathogen (Labbé and Saleh, 2008). A possible hypothesis that needs future experimental validation would be that the hypervirulent LI strains prevent or delay host cell death by continuing to replicate without activating the immune system (McDougal and Sauer, 2018). LII and LIII strains, in contrast, might therefore induce host cell death faster, eliminating potential replication niches by inadvertently activating the immune systems leading to their clearance or slower growth. LLO, phospholipases PlcA and PlcB, and the metalloprotease Mpl are critical for *L. monocytogenes* infection establishment by facilitating vacuole or phagosome escape to gain access to the nutrient-rich host cell cytosol where the bacteria replicate (Camilli et al., 1993; Camejo et al., 2009). Reduced or absence of PI-PLC activity observed in some strains, especially those from LIII, might have therefore contributed to the reduced virulence as well as growth and survival within the zebrafish embryos. Furthermore, differences in InlC and ActA, proteins critical for cell to cell spread of *L. monocytogenes*, might also have contributed to the observed growth and survival differences.

Peptidoglycan alteration through deacetylation of *N*-acetylglucosamine residues and OatA mediated *O*-acetylation of *N*-acetylmuramic acid that converts the sugar backbone into a poor lysozyme substrate constitutes one of *L. monocytogenes* innate immunity evading strategies (Aubry et al., 2011; Cossart, 2011; Rae et al., 2011). Consistent with this, mutants of the peptidoglycan *N*-deacetylase PdgA, which is also involved in this process, are extremely sensitive to lysozyme and severely impaired in growth within mice (Boneca et al., 2007; Rae et al., 2011). In agreement, the more virulent LI strains that survived and grew better in the zebrafish embryos than all other lineages also demonstrated increased lysozyme tolerance. In addition, two variants of the same strain isolated 5 years apart showed two opposing phenotypes (Muchaamba et al., 2020). N843_15, the later isolate, was resistant, whereas the first isolate N843_10 was very sensitive to lysozyme. This increased tolerance could have contributed to the persistence of N843_15 within the infected prosthetic joint. Increased lysosome tolerance for some strains, however, was not always correlated with enhanced virulence, growth, and survival within the infected zebrafish embryo host. Although the number of examined strains is small, we found that LIII strains, despite their higher lysosome stress tolerance than LII strains, had lower virulence and growth within zebrafish embryos. Serotype-associated differences were observed between CC9 strains. The less virulent serotype 1/2c strains displayed higher lysosome tolerance than 1/2a strains from the same clonal complex. Lineage-associated SNP-induced aa changes in PgdA with predicted lineage-based protein structural differences were observed that might also alter PdgA enzymatic activity in a genetic background-based manner leading to such differences in lysozyme tolerance as well as survival and growth within the infected zebrafish embryo host. Furthermore, possible differential *pgdA* expression between study strains could be yet another explanation for the observed lysosome stress tolerance differences. The serotype-based trends between CC9 strains might also be due to cell wall structure differences that also form the basis for serotype classification. Interestingly, the recently described *L. monocytogenes* serotype 4h strains’ hypervirulence is in part aided by their unique WTA structure, which is essential for resistance to antimicrobial peptides, bacterial invasion, and virulence (Yin et al., 2019). Other mechanisms, such as D-alanylation of lipoteichoic acid polymers (through DltABCD proteins), lysinylation of plasma membranes (through MprF), and WTA glycosylation (through GtcA), might play a more important role than peptidoglycan *N*-deacetylation for evasion of the innate host defenses and might contribute to the observed differences in the zebrafish infection model (Carvalho et al., 2014).

Genotype and strain-specific virulence differences could in part be influenced by differences in nutrient acquisition and metabolism strategies or pathways utilized inside the host. Transcriptome-based interrogation of basal metabolism under conditions simulating host conditions showed that lipid and phosphate metabolism was highly enriched in CC1, CC2, CC4, and CC6 strains, whereas the metabolism of aa and related molecules was highly enriched in CC9 and CC121 strains (Lee et al., 2019). We previously also demonstrated intracellular C-source differential metabolism, including pyruvic acid, maltose, inosine, and thymidine among listeriosis outbreak-associated *L. monocytogenes* strains (Muchaamba et al., 2019). Varied distribution of metabolic pathways such as LIPI-4-encoded cellobiose-type phosphotransferase systems, which are linked to increased invasion and neural and placental listeriosis, might also contribute to nutrient utilization and virulence differences within the host (Maury et al., 2016). SNP-induced structural differences observed in Hpt, an important virulence factor facilitating intracellular growth by sugar–phosphate transportation (Joseph et al., 2006), might alter these functions in a lineage-based manner contributing to lineage-based bacteria growth profile differences as observed in the zebrafish embryos.

Strain-specific and/or intra-clonal complex differences such as those observed in CC1 and CC9 could have been due to strain-specific SNPs, InDels, and gene presence–absence patterns. For instance, CC1 strain N12-1996 has a truncated ArgA protein, whereas the avirulent CC9 serotype 1/2c and 3c strains encode truncated InlA proteins. InlA is important for cell invasion and crossing the intestine–blood barrier (Nightingale et al., 2005b; Cossart, 2011; Drolia and Bhunia, 2019). In our study, we used the intravenous route for infection; interestingly, strains with InlA mutations were avirulent or hypovirulent, indicating InlA’s importance post-circulatory system access. Similar to our findings, other studies have also seen an enrichment of InlA truncation in CC121 as well as serotype 1/2c and 3c strains suggestive of an evolutionary predisposition of these strains to PMSC in *inlA* (Van Stelten et al., 2010; Gelbíčová et al., 2015, 2016; Su et al., 2019). *L. monocytogenes* N05-195 carries several PMSC in genes important for virulence and stress tolerance, including *prfA* and *inlA* explaining its virulence loss and increased stress sensitivity. It can be inferred that this strain might have been exposed to environmental conditions that promote the occurrence of SNPs and PMSC. Collectively, our findings on *L. monocytogenes* serotype 1/2c strains offer further evidence on why these strains are rarely involved in human listeriosis cases. Moreover, variable distribution patterns, SNPs, and InDels in other internalins, including InlC, InlD, InlJ, InlL, InlF, and InlG, could have contributed to the observed virulence differences.

Listeriosis cases due to CC121 strains encoding truncated InlA proteins have been reported (Gelbíčová et al., 2015; Maury et al., 2016; Su et al., 2019). Two of the four hypovirulent CC121 strains examined in our study were also clinical case isolates and carried truncated InlA. These observations are suggestive that unlike the situation with PrfA, truncation of InlA is not an all or non-event regarding virulence. In cases of high contamination levels and/or highly susceptible hosts, *L. monocytogenes* strains carrying such truncated InlA can potentially have severe negative health impacts. Such observations might justify a zero-tolerance approach for *L. monocytogenes* regardless of hypovirulence markers, especially for food destined for high-risk individuals.

Through the function of transport systems such as the Sec system, virulence factors or their precursors are delivered to their site of action on the cell surface or extracellular environment (Desvaux and Hebraud, 2006; Carvalho et al., 2014). Mutants of these systems or their components, such as SecA2 and SecDF, have been shown to display reduced virulence (Lenz et al., 2003; Burg-Golani et al., 2013). Protein sequence altering SNPs and InDels detected in some of these genes might therefore have functional consequences altering virulence factor secretion contributing to overall virulence phenotypic differences observed between strains in this study.

Gene expression regulation, posttranscriptional regulation, posttranslational regulation, and/or posttranslational modification differences, which we could not predict by genome analysis alone, could also contribute to the overall phenotypic differences observed. Differences in the presence and content of the restriction modification system and DNA methylation enzymes observed could potentially have contributed to these phenotypic differences at this level. For instance, CC4 and CC8 strains contained clone-specific phase variation RMS; some of these systems might be responsible for the “atypical” phenotypes observed with strains N12-2747 (CC1), N12-0794 (CC4), and N12-1387 (CC6), which displayed very low hemolysis and PI-PLC activity but were hypervirulent against the zebrafish embryos. Such shifts between phenotypic forms are also observed in other bacteria such as *Streptococcus pneumoniae* (Manso et al., 2014). A previous study using a neonatal rat-based *Listeria* meningitis model and an *L. monocytogenes* CC4 strain carrying a similar type I RMS as N12-0794 showed that phase variations linked to these systems result in altered disease severity outcomes (Zbinden et al., 2020). These phase variable epigenetic modifications might allow some strains to finely tune their virulence gene expression, only expressing them when specific host conditions are encountered. These phenotypically atypical *L. monocytogenes* strains can pose diagnostic challenges, potential being misclassified as non-pathogenic *Listeria*, thereby acting as Trojan horses exposing consumers to hypervirulent strains.

Osmotic and pH stress exposure in food has been postulated to increase *L. monocytogenes* virulence by priming the bacteria against digestive tract stresses (Garner et al., 2006; Barbosa et al., 2012; Pettersen et al., 2018). Strains examined differed in NaCl osmotic stress tolerance, with LI, serotype 4b, CC2 and CC4 strains being more osmotolerant, whereas LII and CC9 strains were most osmosensitive. We have previously observed similar osmotic stress tolerance variation among listeriosis outbreak strains (Muchaamba et al., 2019). LI, serotype 4b strain of our study displayed the highest tolerance to osmotic stress. Interestingly some of the osmotic stress results observed mirrored virulence phenotypic trends. CC2 and CC4 strains highly resistant to osmotic stress were also hypervirulent, whereas hypovirulent CC9 strains were the most sensitive to osmotic stress.

Salt alone or in combination with other hurdle technologies is often used as a general preservative and an antibacterial agent because of its inhibitory effects on bacterial growth in RTE food (Desmond, 2006). *L. monocytogenes* efficiently adapts and sometimes proliferates, despite exposure to low temperatures, low pH, and elevated salt (NaCl) concentrations, conditions used in preserving RTE food products (Soni et al., 2011; Burgess et al., 2016; Wiktorczyk-Kapischke et al., 2021). *L. monocytogenes* ability to accumulate potassium and compatible solutes, such as glycine betaine, carnitine, proline, and trehalose, is important for osmotic stress tolerance (Sleator and Hill, 2002; Gandhi and Chikindas, 2007; Soni et al., 2011). Compatible solutes also function as cryoprotectants increasing cold stress tolerance (Angelidis and Smith, 2003; Wemekamp-Kamphuis et al., 2004; Gandhi and Chikindas, 2007; Singh et al., 2011). These solutes are accumulated through the function of various transport protein systems, including BetL, Gbu, OpuC, ProBA, and TreA (Burgess et al., 2016). The Na/K^+^ antiporter and its regulator are key for potassium accumulation, which is an initial critical response to osmotic stress. Protease HtrA and ClpP, ClpC (an ATPase), the LisRK two-component regulatory system, and Csps also play critical roles against such stressors (Burgess et al., 2016; Muchaamba et al., 2021a). Most of the genes encoding these proteins are under the control of σ*^B^* (Chaturongakul et al., 2008; O’Byrne and Karatzas, 2008). Strain and genetic background-associated SNPs were observed in these proteins, some of which resulted in significant structural changes. These might have contributed to the lineage and strain-specific differences in *L. monocytogenes* osmotic stress sensitivity observed. Our data agree with previous studies demonstrating that LI and LIII strains were more osmotolerant than LII strains (Bergholz et al., 2010; Hingston et al., 2017; Muchaamba et al., 2019). Such strain-specific differences in osmotic tolerance need to be considered when performing food challenge tests on low water activity food, bearing in mind that selecting strains that are too sensitive will underestimate the risk. At the same time, selecting the highly tolerant strains, which might be seen by many as the most appropriate approach, might overestimate the risk. The best would probably be to include a diverse set of strains for such studies.

Cross-protection between stressors has been reported for many hurdle techniques against *L. monocytogenes* (Gandhi and Chikindas, 2007; Bergholz et al., 2012; Begley and Hill, 2015; Muchaamba et al., 2021a; Wu et al., 2021). We observed that low salt concentration promotes adaptation of *L. monocytogenes* to cold stress as indicated by faster induction of growth. Such observations are suggestive of enhanced adaptation to cold stress in the presence of low salt concentration, possible as a consequence of dual activation of stress response systems involved in both cold and salt stress. Osmotic stress exposure-associated promotion of *L. monocytogenes* cold stress adaptation has implications for practical food microbial control measures. Combined or sequential exposure of *L. monocytogenes* cells to these two stresses in food environments might inadvertently induce cross-protection responses. As such, current efforts to reduce NaCl levels used as preservatives in food to improve human health will also be mitigatory against cold growth-promoting effects of salt exposure on *L. monocytogenes* (Muchaamba et al., 2021b; Rysová and Šmídová, 2021).

Control of *L. monocytogenes* continues to challenge the food industry through tolerance of common disinfectants and decontamination procedures (Ebner et al., 2015; Bucur et al., 2018; Wiktorczyk-Kapischke et al., 2021). Due to this resistance and biofilm production ability, once introduced in a facility, the chances of *L. monocytogenes* persistence for long periods are high (Orsi et al., 2008; Ferreira et al., 2014; Leong et al., 2014; Buchanan et al., 2017; Stoller et al., 2019; Kragh et al., 2020). Our study strains also varied in tolerance to the widely used quaternary ammonium compound BC. Two outbreak and five sporadic clinical case-associated strains demonstrated BC resistance. Possessing such a phenotypic trait might have aided their survival and transition in the food-processing environment facilitating food product contamination resulting in outbreaks and sporadic listeriosis cases. As expected, the CC121 strains carried BC resistance genes. However, mechanisms responsible for increased resistance for most BC-tolerant strains, including the two outbreak strains (N1546 and Lm3136), are not yet established.

Genome-wide sequence comparison of the strains targeting virulence and stress tolerance-associated genes suggests that a few genetic differences and variations in gene regulation and expression between the strains are largely responsible for the phenotypic differences observed. Some of these genetic differences in virulence, nutrient utilization, and stress resilience-associated genes followed a phylogeny-dependent trend. SSI-1 islet has been previously shown to be a feature of *L. monocytogenes* CC7 and CC8 strains associated with persistence in food-processing plants, but it is also found in sporadic environmental strains (Knudsen et al., 2017). A similar picture was observed in this study; SSI-1 was identified in all CC7, CC8, and CC9 strains. Extensive SNP, InDel, and protein structure analysis identified significant differences affecting important virulence and stress tolerance-associated proteins. As these genetic background-associated structural changes occurred in several proteins linked to stress tolerance and virulence, their individual and/or sum-total effects might have significantly contributed to the observed phenotypic differences. Overall, our observation suggests that the differences observed in virulence and stress tolerance capacity among the examined strains might involve variation in a few genes and/or differences in overall gene regulation and expression between strains or genetic subgroups. For instance, previous studies have suggested that *L. monocytogenes* lysozyme resistance is mainly through the regulation, not the acquisition, of cell wall-modifying enzymes (Burke et al., 2014). To unearth and corroborate the effects of such differences, proteomic, transcriptomic, and mutational approaches will need to be applied in the future to validate most of the hypotheses put forward in this study.

Although interesting phenotypic differences and possible reasons for the observations have been reported, at this stage, some of the possible mechanisms for these variabilities in *L. monocytogenes* are still based on extrapolations centered on knowledge derived from other organisms. Therefore, these remain to be validated experimentally. However, this study provides a strong basis for future work on *L. monocytogenes* genetic background-based variations in virulence and stress tolerance. Pertinent questions, such as the role of lineage-specific SNPs and InDels in this phenotypic variability, need to be answered. As with many studies that use virulence models (Liu et al., 2007), the design of the current study is subject to limitations. Zebrafish embryo infection model drawbacks such as the applied experimental temperature (28°C) and intravenous infection route used that bypasses the gastrointestinal tract passage must be considered in interpreting or extrapolating our findings. In addition, some *L. monocytogenes* isolates were seen not to grow in zebrafish (Menudier et al., 1996). Nonetheless, several studies support the use of zebrafish as an *L. monocytogenes* virulence model (Levraud et al., 2009; Nowik et al., 2015; Shan et al., 2015; Torraca and Mostowy, 2018). Furthermore, we also found that, as expected, strains with truncated virulence factors such as PrfA (N843_10, N843_15, and N05-195), PlcB (H34), InlA (N12-0486, N11-1837, N13-0001, and N11-1514), and InlC (WLSC1019 and WLSC1020) are either avirulent or hypovirulent further supporting the use of this model.

## Conclusion

Our study has further shown stratification of *L. monocytogenes* virulence and stress tolerance at the lineage, serotype, and CC levels consistent with the view that strains of this foodborne pathogen are not created equal. Our data support the notion that *L. monocytogenes* LI gravitates toward being a human host-adapted lineage, whereas LII and LIII appear to be environmentally adapted lineages (Nightingale et al., 2005a). Taken together, these findings strengthen the need for functional analysis to be done using more clinically relevant genetic backgrounds such as CC1, CC2, CC4, and CC6. *L. monocytogenes* reference strain EGDe previously used in many experiments belongs to CC9, which showed inferior virulence and stress tolerance to strains belonging to the genetic backgrounds mentioned earlier. Overall, such phenotypic and genotypic variability can be a selective advantage within-host, natural, and food-associated environments allowing for increased survival chances in food and expanded colonized niche range in external environments for some *L. monocytogenes* strains. This knowledge can be utilized in the future to redefine risks posed by different *L. monocytogenes* strains and in development of appropriate policy that does not over or underestimate the threats posed by each different strain.

## Data Availability Statement

The datasets presented in this study can be found in online repositories. The names of the repository/repositories and accession number(s) can be found in the article/Supplementary Material.

## Ethics Statement

Ethical review and approval was not required for the animal study because the maximum age of the embryos during experimentation was 5 days post fertilization, therefore, no license was required from the cantonal veterinary office in Switzerland as such embryos will not have yet reached the free-feeding stage. All protocols used in this study adhered to the standards of the “Ordinance on laboratory animal husbandry, the production of genetically modified animals and the methods of animal experimentation; Animal Experimentation Ordinance” (SR 455.163, April 12, 2010), Swiss Federal Food Safety and Veterinary Office (FSVO/BLV). Internationally recognized standards and Swiss legal ethical guidelines for the use of fish in biomedical research were followed for all animal protocols. All the experiments were approved by the local authorities (Veterinäramt Zürich Tierhaltungsnummer 150).

## Author Contributions

FM and TT designed the study. TT and RS supervised the study. FM performed all the experiments, analyzed the data, wrote the first manuscript draft, and did final editing and review consolidation. AE and FM performed the zebrafish embryo microinjection experiments. FM and MS conducted bioinformatic analyses. MS, RS, and TT analyzed the data and edited the manuscript. All authors have read and agreed to the published version of the manuscript.

## Conflict of Interest

The authors declare that the research was conducted in the absence of any commercial or financial relationships that could be construed as a potential conflict of interest.

## Publisher’s Note

All claims expressed in this article are solely those of the authors and do not necessarily represent those of their affiliated organizations, or those of the publisher, the editors and the reviewers. Any product that may be evaluated in this article, or claim that may be made by its manufacturer, is not guaranteed or endorsed by the publisher.

## Footnotes

1
blast.ncbi.nlm.nih.gov/Blast.cgi

2
https://bigsdb.pasteur.fr/listeria

3
http://www.sbg.bio.ic.ac.uk/~missense3d/
