# Supplementary material for: Different Shades of Listeria monocytogenes: Strain, Serotype, and Lineage-Based Variability in Virulence and Stress Tolerance Profiles
Source: Front Microbiol. 2022 Jan 4;12:792162. doi: 10.3389/fmicb.2021.792162 (PMC8764371; doi:10.3389/fmicb.2021.792162)
Supplement: Supplementary file 1 [file Presentation_1.pptx]

## Slide 1
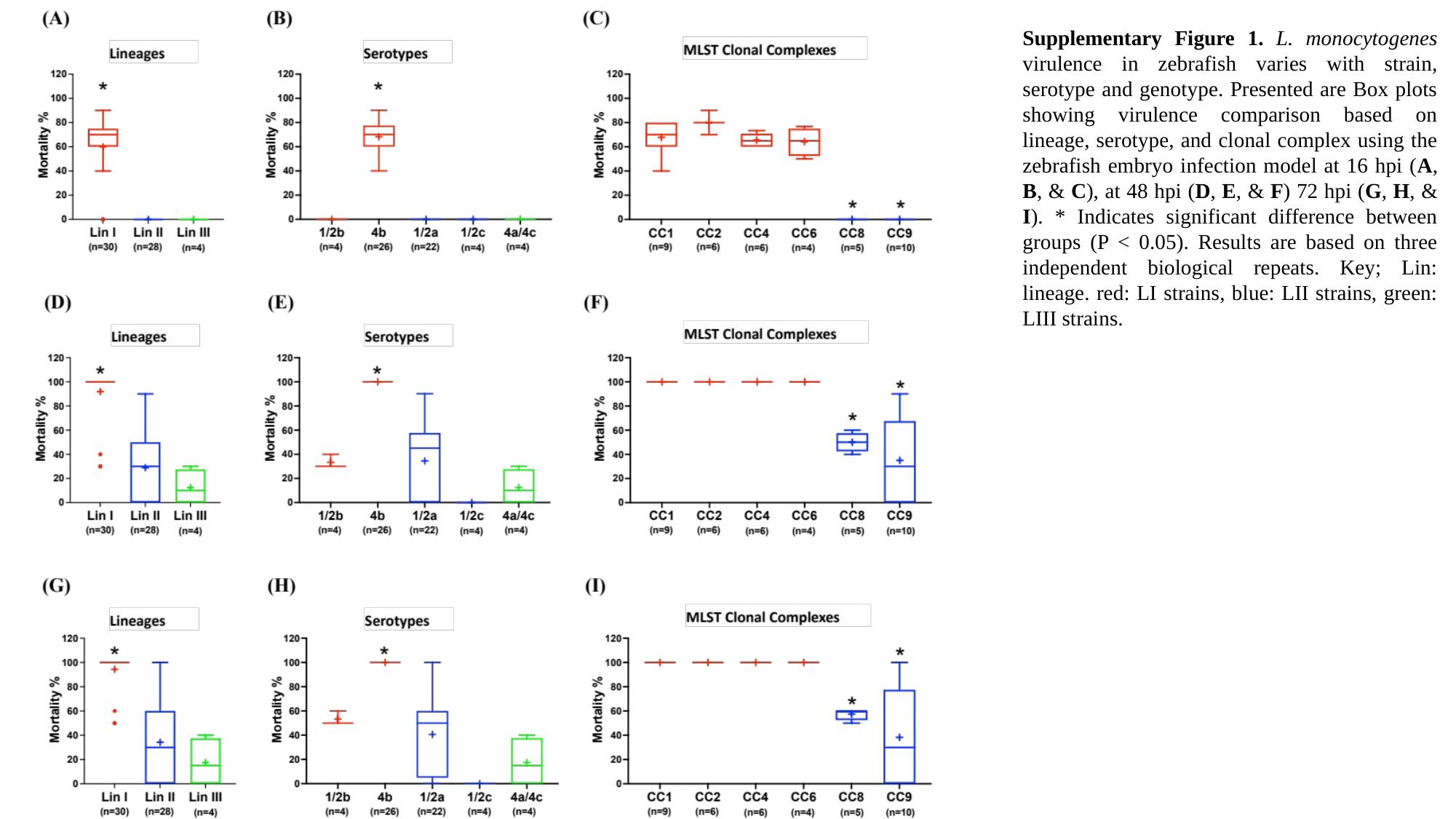

Supplementary Figure 1. L. monocytogenes virulence in zebrafish varies with strain, serotype and genotype. Presented are Box plots showing virulence comparison based on lineage, serotype, and clonal complex using the zebrafish embryo infection model at 16 hpi (A, B, & C), at 48 hpi (D, E, & F) 72 hpi (G, H, & I). * Indicates significant difference between groups (P < 0.05). Results are based on three independent biological repeats. Key; Lin: lineage. red: LI strains, blue: LII strains, green: LIII strains.

## Slide 2
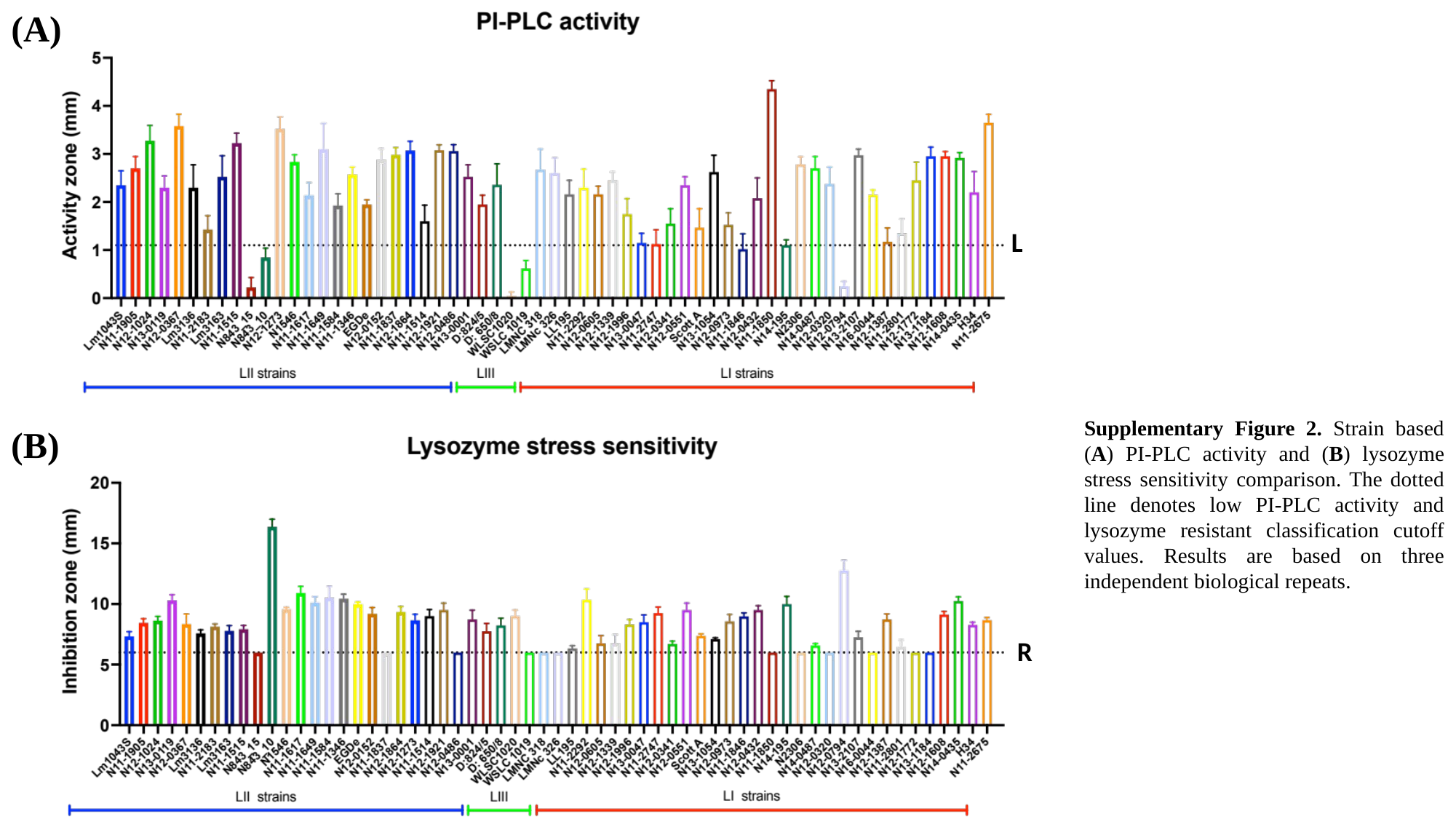

(A)
L
(B)
R
Supplementary Figure 2. Strain based (A) PI-PLC activity and (B) lysozyme stress sensitivity comparison. The dotted line denotes low PI-PLC activity and lysozyme resistant classification cutoff values. Results are based on three independent biological repeats.

## Slide 3
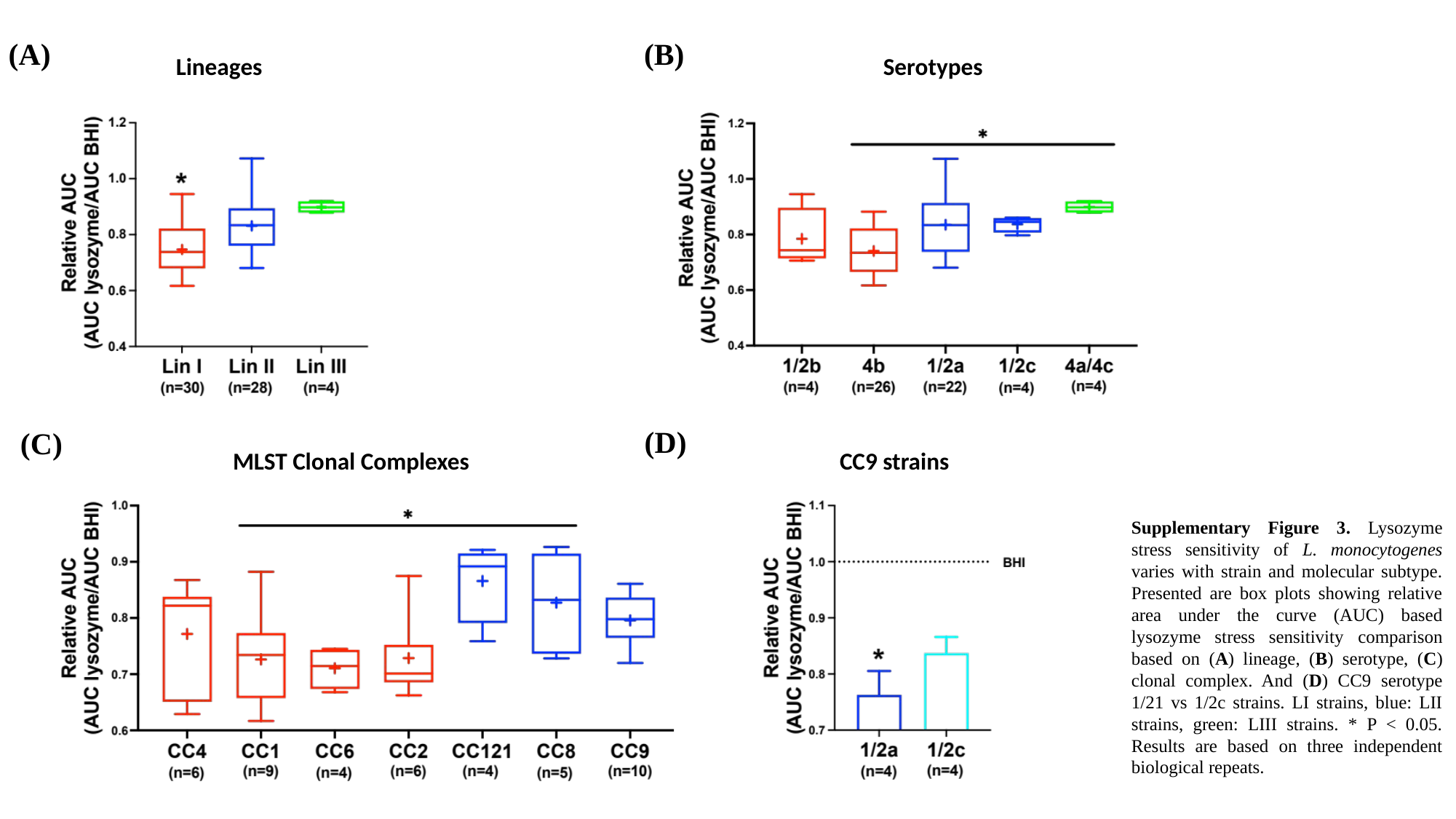

(A)
(B)
Lineages
Serotypes
(D)
(C)
CC9 strains
MLST Clonal Complexes
Supplementary Figure 3. Lysozyme stress sensitivity of L. monocytogenes varies with strain and molecular subtype. Presented are box plots showing relative area under the curve (AUC) based lysozyme stress sensitivity comparison based on (A) lineage, (B) serotype, (C) clonal complex. And (D) CC9 serotype 1/21 vs 1/2c strains. LI strains, blue: LII strains, green: LIII strains. * P < 0.05. Results are based on three independent biological repeats.

## Slide 4
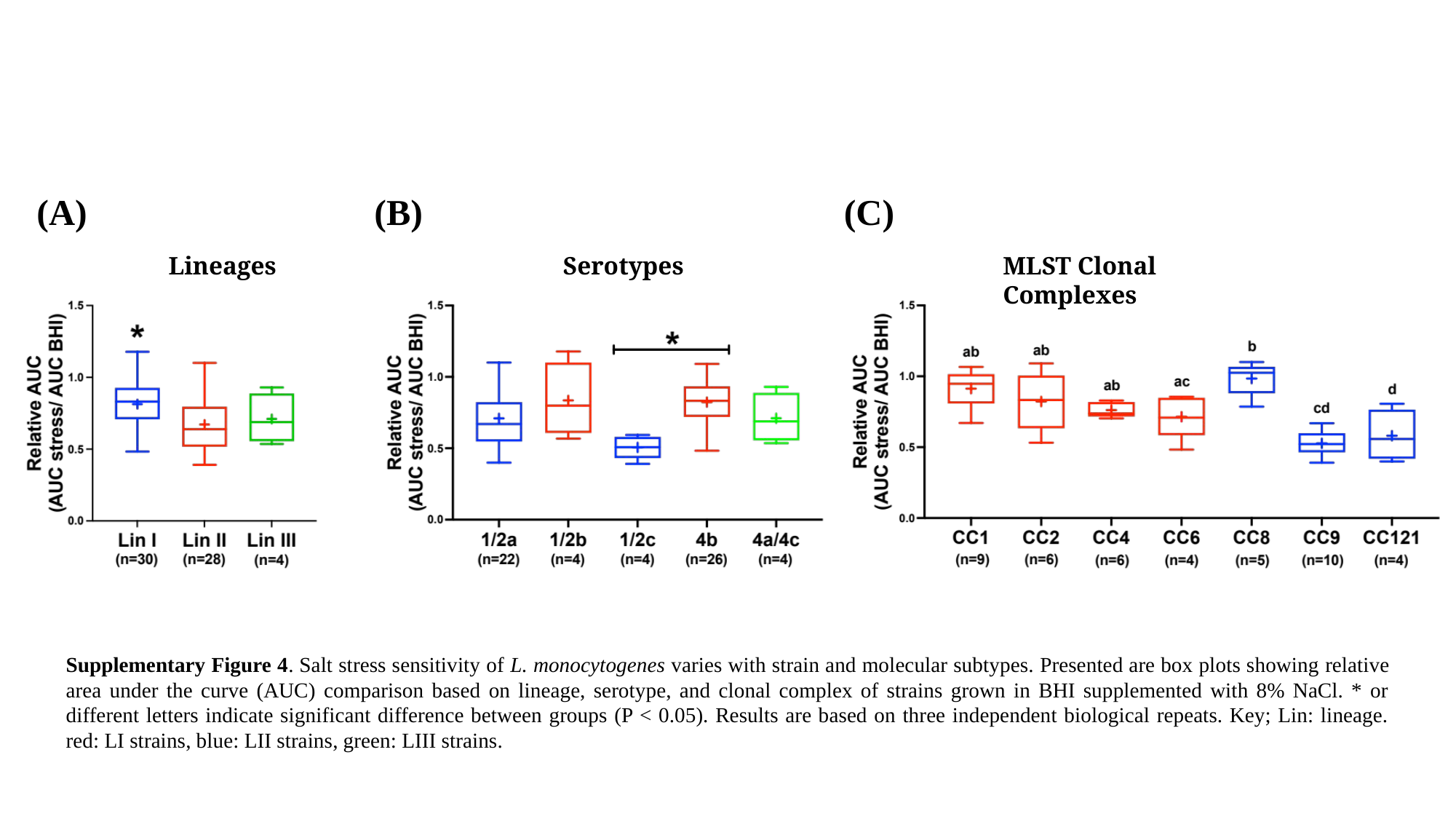

(A)
(B)
(C)
Lineages
Serotypes
MLST Clonal Complexes
Supplementary Figure 4. Salt stress sensitivity of L. monocytogenes varies with strain and molecular subtypes. Presented are box plots showing relative area under the curve (AUC) comparison based on lineage, serotype, and clonal complex of strains grown in BHI supplemented with 8% NaCl. * or different letters indicate significant difference between groups (P < 0.05). Results are based on three independent biological repeats. Key; Lin: lineage. red: LI strains, blue: LII strains, green: LIII strains.

## Slide 5
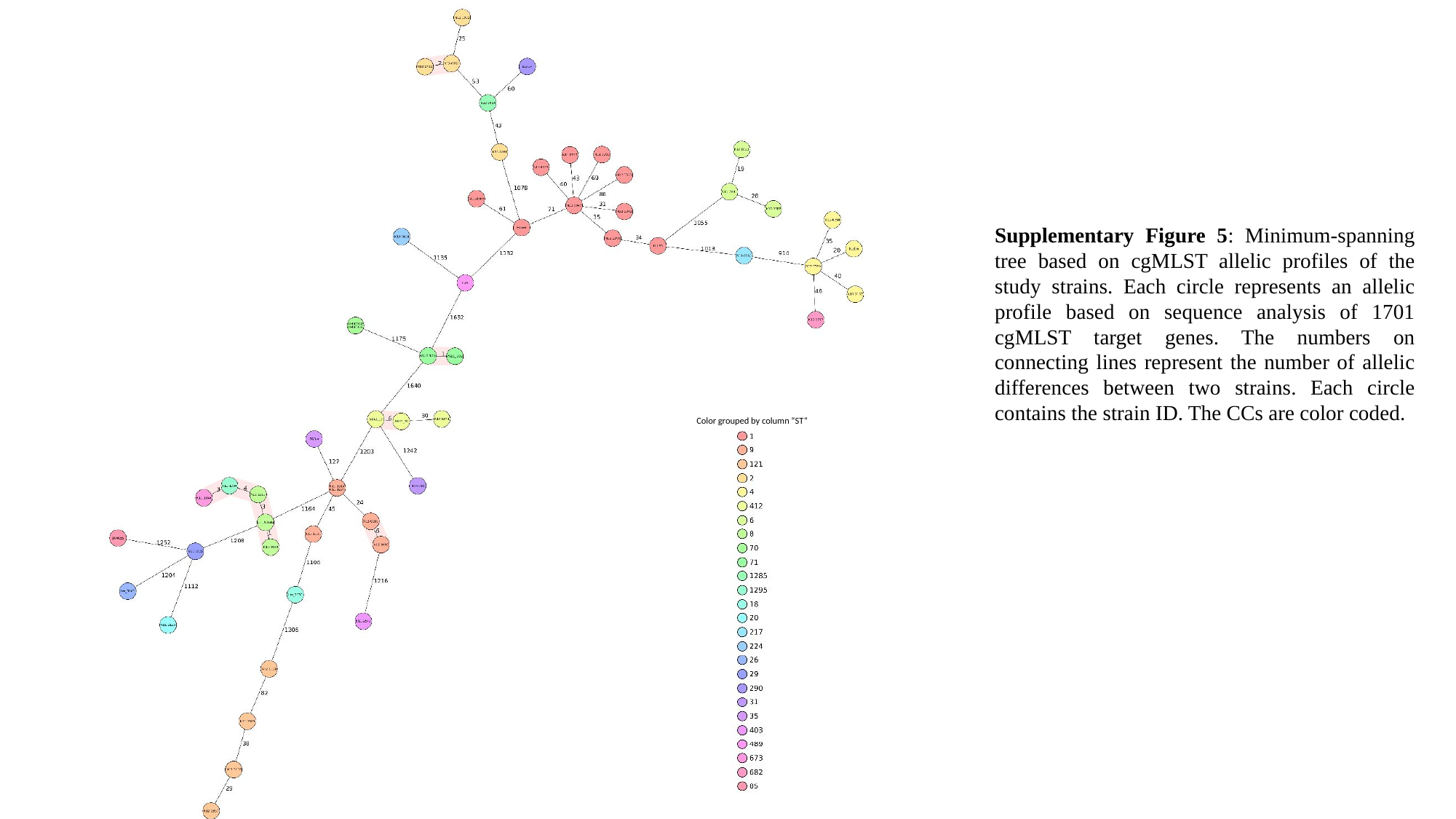

Color grouped by column “ST”
Supplementary Figure 5: Minimum-spanning tree based on cgMLST allelic profiles of the study strains. Each circle represents an allelic profile based on sequence analysis of 1701 cgMLST target genes. The numbers on connecting lines represent the number of allelic differences between two strains. Each circle contains the strain ID. The CCs are color coded.

## Slide 6
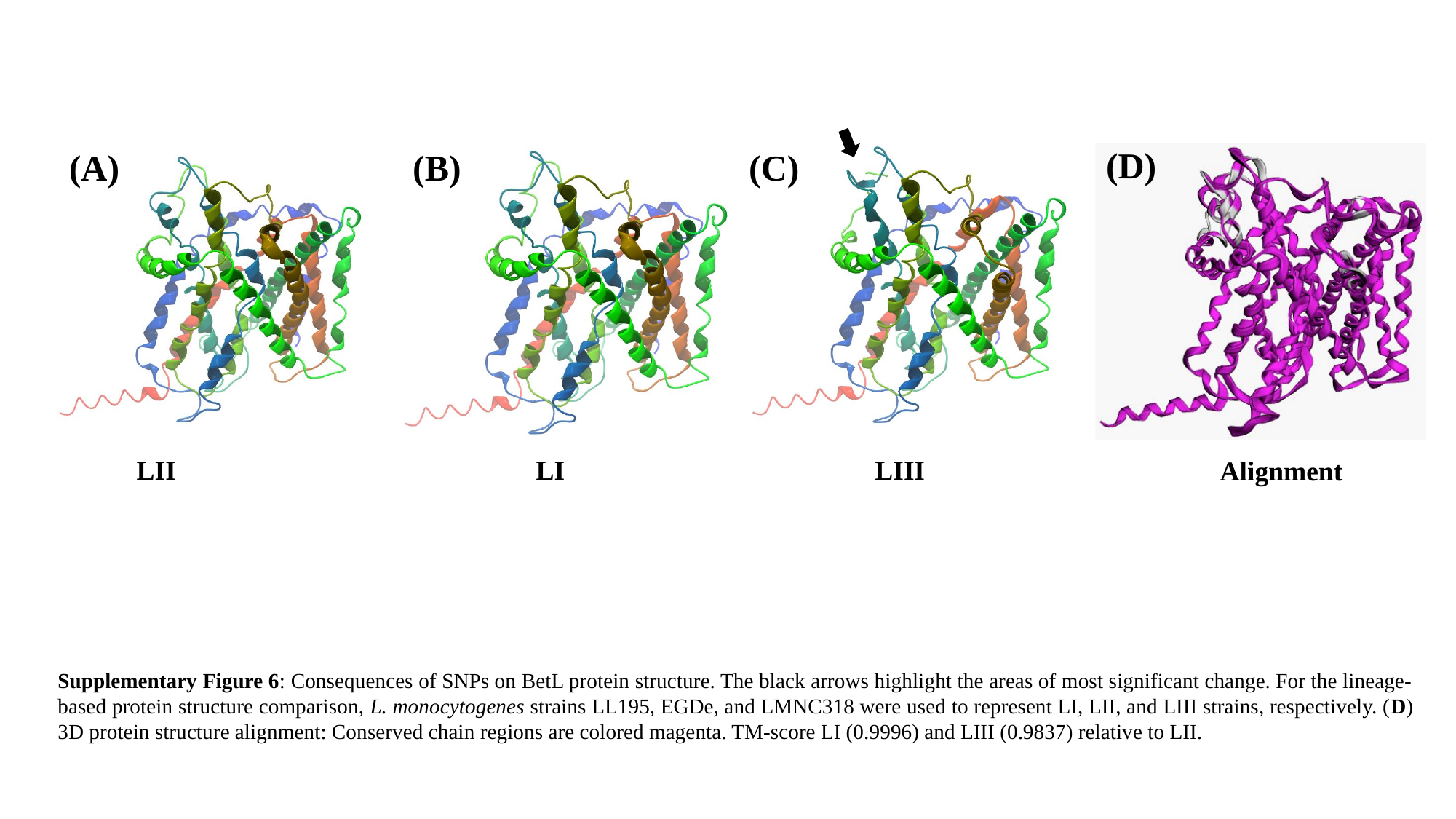

(D)
(B)
(A)
(C)
LII
LI
LIII
Alignment
Supplementary Figure 6: Consequences of SNPs on BetL protein structure. The black arrows highlight the areas of most significant change. For the lineage-based protein structure comparison, L. monocytogenes strains LL195, EGDe, and LMNC318 were used to represent LI, LII, and LIII strains, respectively. (D) 3D protein structure alignment: Conserved chain regions are colored magenta. TM-score LI (0.9996) and LIII (0.9837) relative to LII.

## Slide 7
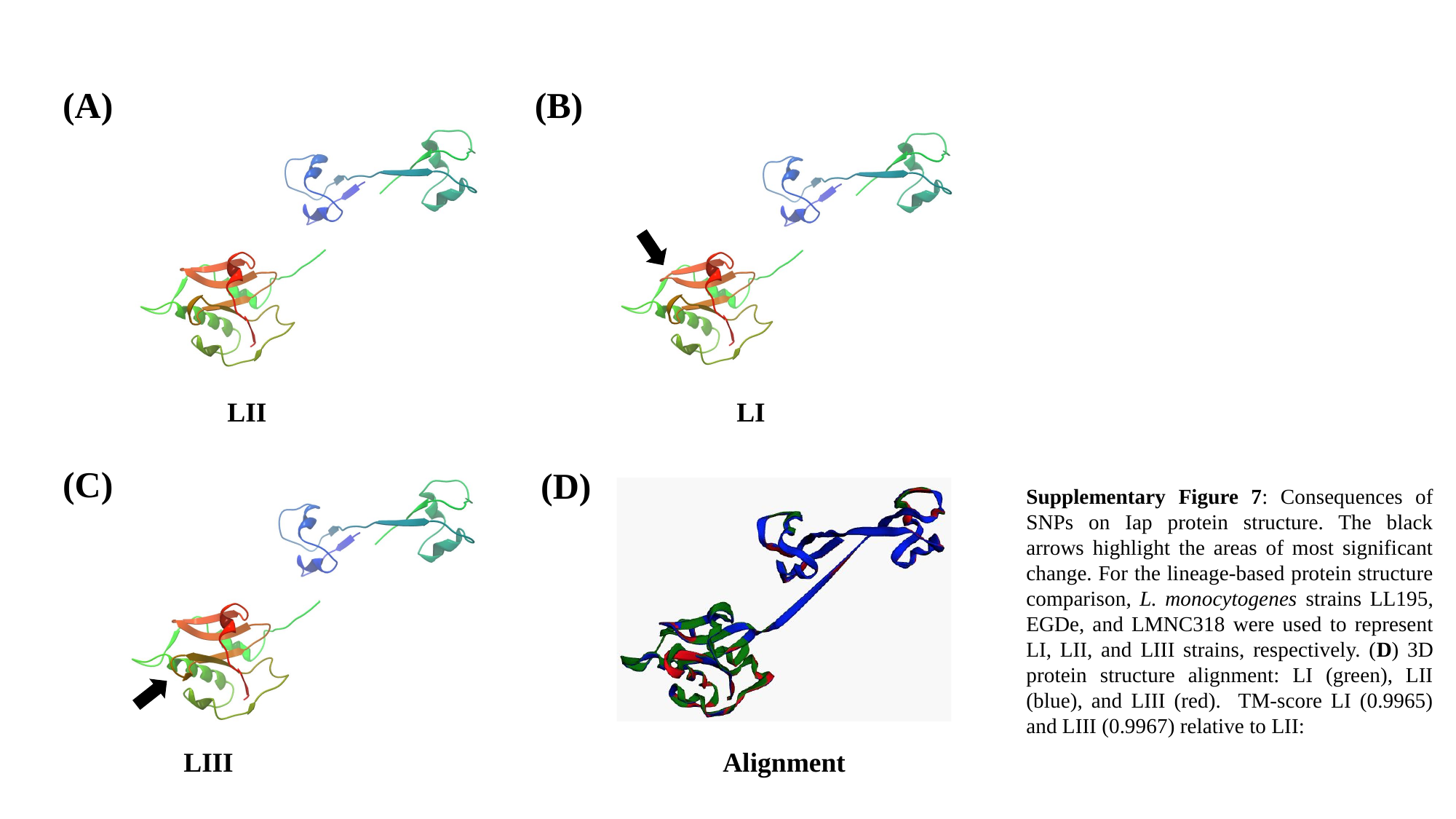

(A)
(B)
LII
LI
(C)
(D)
LIII
Alignment
Supplementary Figure 7: Consequences of SNPs on Iap protein structure. The black arrows highlight the areas of most significant change. For the lineage-based protein structure comparison, L. monocytogenes strains LL195, EGDe, and LMNC318 were used to represent LI, LII, and LIII strains, respectively. (D) 3D protein structure alignment: LI (green), LII (blue), and LIII (red). TM-score LI (0.9965) and LIII (0.9967) relative to LII:

## Slide 8
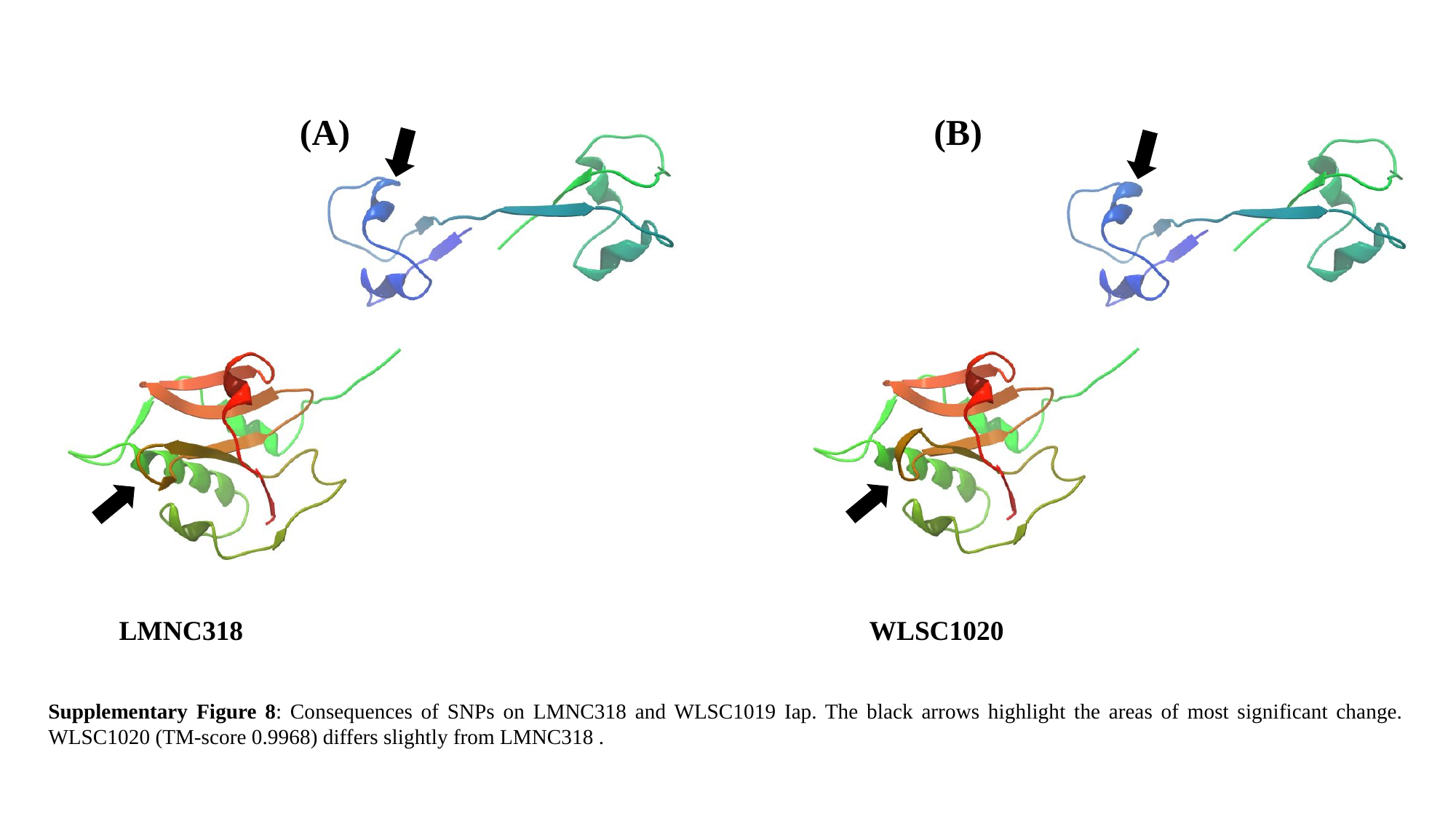

(A)
(B)
LMNC318
WLSC1020
Supplementary Figure 8: Consequences of SNPs on LMNC318 and WLSC1019 Iap. The black arrows highlight the areas of most significant change. WLSC1020 (TM-score 0.9968) differs slightly from LMNC318 .

## Slide 9
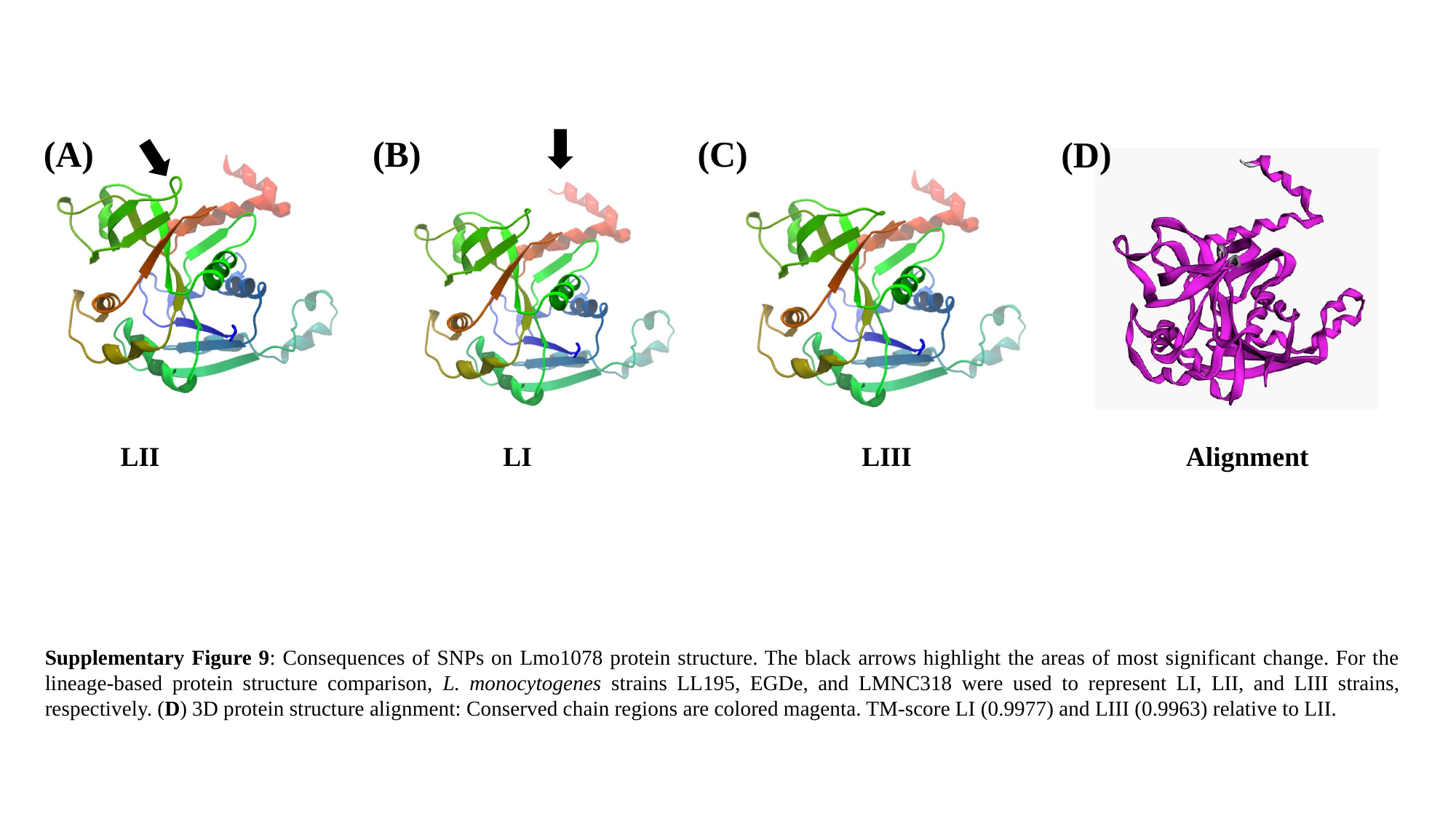

(A)
(B)
(C)
(D)
LII
LI
LIII
Alignment
Supplementary Figure 9: Consequences of SNPs on Lmo1078 protein structure. The black arrows highlight the areas of most significant change. For the lineage-based protein structure comparison, L. monocytogenes strains LL195, EGDe, and LMNC318 were used to represent LI, LII, and LIII strains, respectively. (D) 3D protein structure alignment: Conserved chain regions are colored magenta. TM-score LI (0.9977) and LIII (0.9963) relative to LII.

## Slide 10
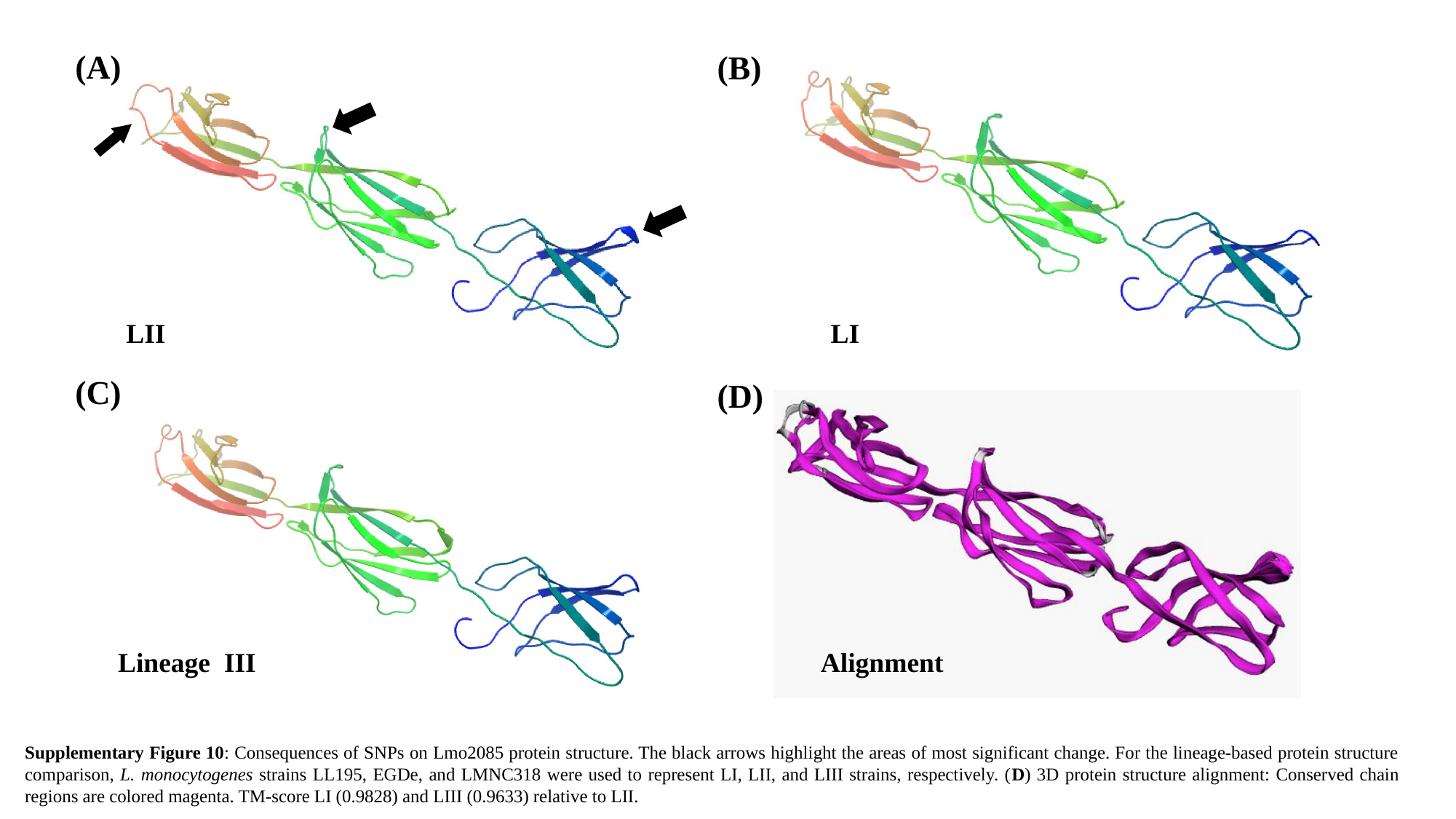

(A)
(B)
LII
LI
(C)
(D)
Lineage III
Alignment
Supplementary Figure 10: Consequences of SNPs on Lmo2085 protein structure. The black arrows highlight the areas of most significant change. For the lineage-based protein structure comparison, L. monocytogenes strains LL195, EGDe, and LMNC318 were used to represent LI, LII, and LIII strains, respectively. (D) 3D protein structure alignment: Conserved chain regions are colored magenta. TM-score LI (0.9828) and LIII (0.9633) relative to LII.

## Slide 11
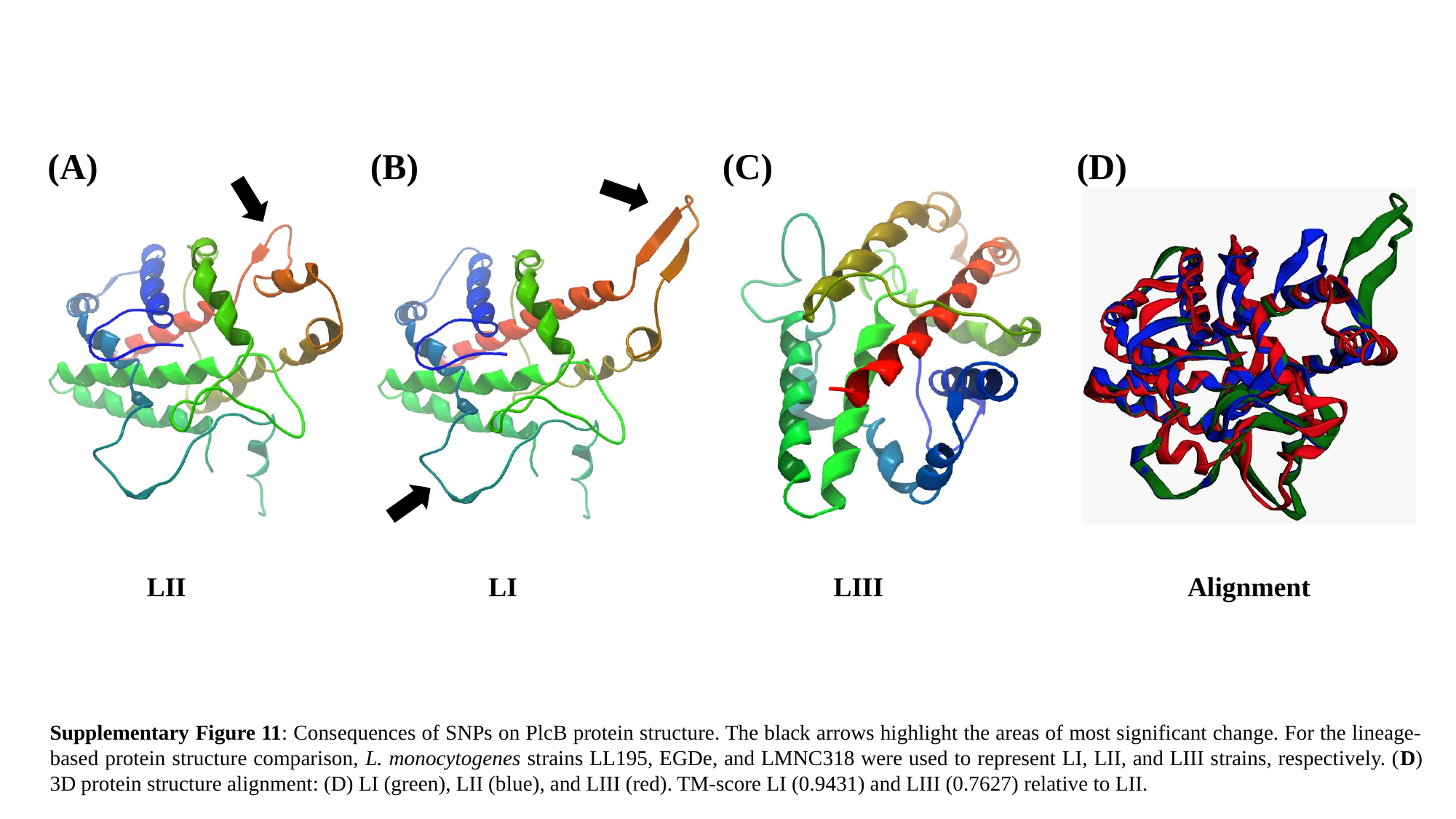

(A)
(C)
(D)
(B)
LII
LI
LIII
Alignment
Supplementary Figure 11: Consequences of SNPs on PlcB protein structure. The black arrows highlight the areas of most significant change. For the lineage-based protein structure comparison, L. monocytogenes strains LL195, EGDe, and LMNC318 were used to represent LI, LII, and LIII strains, respectively. (D) 3D protein structure alignment: (D) LI (green), LII (blue), and LIII (red). TM-score LI (0.9431) and LIII (0.7627) relative to LII.

## Slide 12
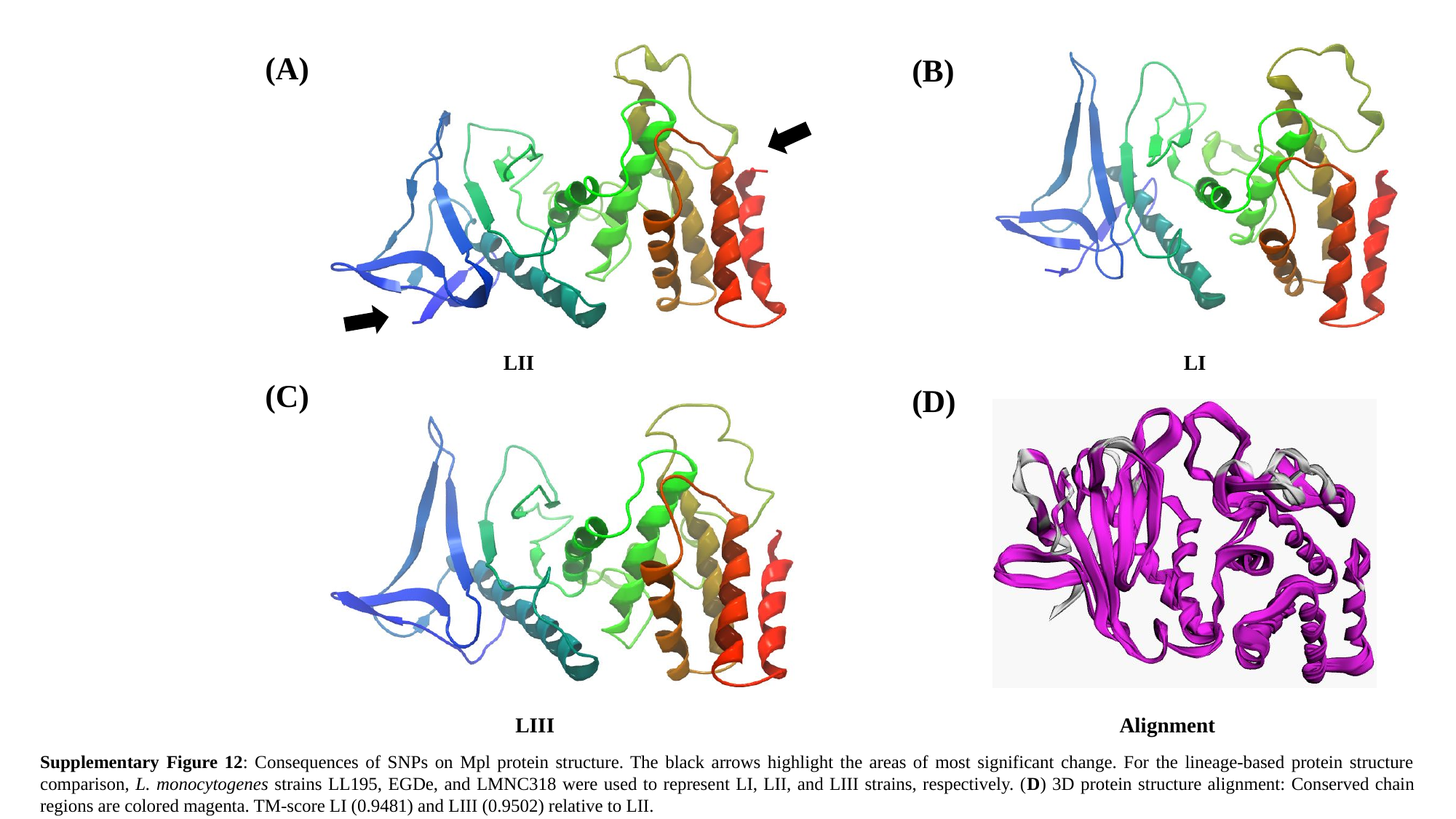

(A)
(B)
LII
LI
(C)
(D)
Alignment
LIII
Supplementary Figure 12: Consequences of SNPs on Mpl protein structure. The black arrows highlight the areas of most significant change. For the lineage-based protein structure comparison, L. monocytogenes strains LL195, EGDe, and LMNC318 were used to represent LI, LII, and LIII strains, respectively. (D) 3D protein structure alignment: Conserved chain regions are colored magenta. TM-score LI (0.9481) and LIII (0.9502) relative to LII.

## Slide 13
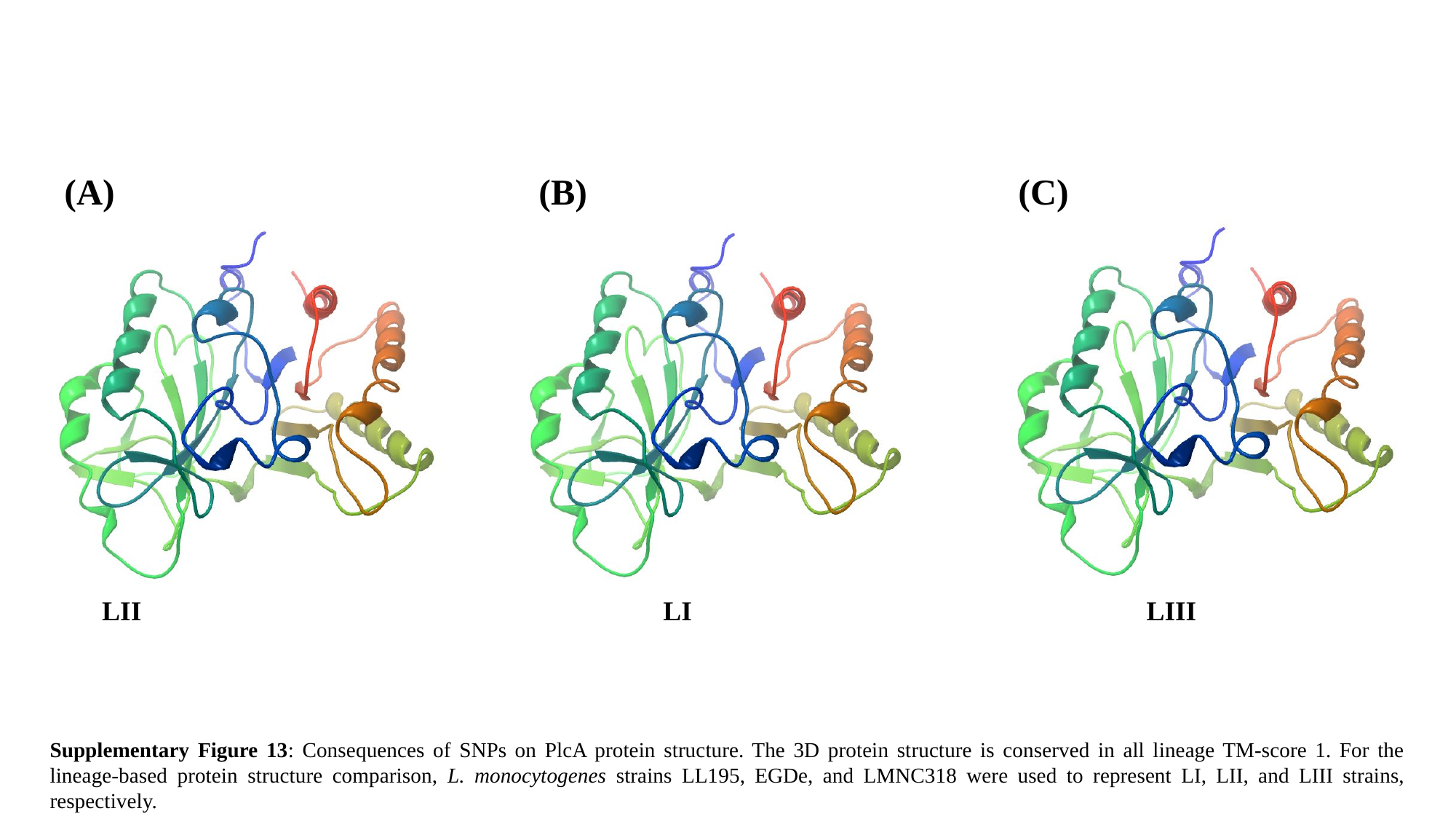

(A)
(B)
(C)
LII
LI
LIII
Supplementary Figure 13: Consequences of SNPs on PlcA protein structure. The 3D protein structure is conserved in all lineage TM-score 1. For the lineage-based protein structure comparison, L. monocytogenes strains LL195, EGDe, and LMNC318 were used to represent LI, LII, and LIII strains, respectively.
