# Supplementary material for: Different Shades of Listeria monocytogenes: Strain, Serotype, and Lineage-Based Variability in Virulence and Stress Tolerance Profiles
Source: Front Microbiol. 2022 Jan 4;12:792162. doi: 10.3389/fmicb.2021.792162 (PMC8764371; doi:10.3389/fmicb.2021.792162)
Supplement: Supplementary file 3 [file Table_1.docx]

**Supplementary Table 1**. Study strains

| **Strain ID** | **Source** | **ST^a^** | **CC^a^** | **Serotypes** | **Lineage** | **Reference** | **Accession number** | **BC MIC (µg/mL)^b^** |
| --- | --- | --- | --- | --- | --- | --- | --- | --- |
| LL195**^cde^** | Vacherin Montd’or cheese | ST1 | CC1 | 4b | I | Bille, 1990 | HF558398 | 3.5 |
| N11-2292 | human listeriosis | ST1 | CC1 | 4b | I | Althaus et al., 2014 | JABYYE000000000 | 3.5 |
| N12-0605 | Meat / Meat product | ST727 | CC1 | 4b | I | Ebner et al., 2015 | JABYXR000000000 | 3.5 |
| N12-1339 | Meat / Meat product | ST746 | CC1 | 4b | I | Ebner et al., 2015 | JABYXM000000000 | 3.5 |
| N12-1996 | Milk / Milk product | ST1 | CC1 | 4b | I | Ebner et al., 2015 | JABYXD000000000 | 3.5 |
| N13-0047 | Milk / Milk product | ST1 | CC1 | 4b | I | Ebner et al., 2015 | JABYWZ000000000 | 3.5 |
| N11-2747 | human listeriosis | ST1 | CC1 | 4b | I | Althaus et al., 2014 | JABYYC000000000 | 3.5 |
| N12-0341 | human listeriosis | ST1 | CC1 | 4b | I | Althaus et al., 2014 | JABYXY000000000 | 4 |
| N12-0551 | human listeriosis | ST1 | CC1 | 4b | I | Althaus et al., 2014 | JABYXU000000000 | 3.5 |
| Scott A | Human listeriosis | ST290 | CC2 | 4b | I | Fleming et al., 1985 | CM001159 | 3.5 |
| N13-1054 | human listeriosis | ST1285 | CC2 | 4b | I | Althaus et al., 2014 | QYED00000000 | 4 |
| N12-0973 | Meat / Meat product | ST2 | CC2 | 4b | I | Ebner et al., 2015 | JABYXO000000000 | 3.5 |
| N11-1846 | Meat / Meat product | ST724 | CC2 | 4b | I | Ebner et al., 2015 | JABYYG000000000 | 3.5 |
| N12-0432 | Meat / Meat product | ST2 | CC2 | 4b | I | Ebner et al., 2015 | JABYXX000000000 | 3.5 |
| N12-0466 | Meat / Meat product | ST2 | CC2 | 4b | I | Ebner *et al*., 2015 | JABYXW000000000 | 3.5 |
| N14-0435 | Milk product | ST3 | CC3 | 1/2b | I | Ebner et al., 2015 | NS | 7 |
| N2306**^cde^** | Ready-to-eat salads | ST4 | CC4 | 4b | I | Stephan et al., 2015 | CP011004 | 3.5 |
| N14-0487 | Plant associated | ST4 | CC4 | 4b | I | Ebner et al., 2015 | NS | 3.5 |
| N12-0320**^e^** | human listeriosis | ST4 | CC4 | 4b | I | Althaus et al., 2014 | JABYXZ000000000 | 4 |
| N12-0794**^e^** | human listeriosis | ST4 | CC4 | 4b | I | Althaus et al., 2014 | JABYXQ000000000 | 3.5 |
| N13-2107 | Meat / Meat product | ST4 | CC4 | 4b | I | Ebner et al., 2015 | QYDL00000000 | 3.5 |
| N12-1772 | Milk / Milk product | ST682 | CC4 | 4b | I | Ebner et al., 2015 | JABYXH000000000 | 3.5 |
| N11-2675 | human listeriosis | ST1063 | CC5 | 1/2b | I | Althaus et al., 2014 | QYHF00000000 | 3.5 |
| N16-0044**^cde^** | Meat pâté | ST6 | CC6 | 4b | I | Althaus et al., 2017 | CP035187 | 3.5 |
| N12-1387 | human listeriosis | ST6 | CC6 | 4b | I | Althaus et al., 2014 | JABYXL000000000 | 3.5 |
| N11-2801 | human listeriosis | ST6 | CC6 | 4b | I | Althaus et al., 2014 | JABYYB000000000 | 3.5 |
| N13-1184 | Meat / Meat product | ST6 | CC6 | 4b | I | Ebner et al., 2015 | NS | 3.5 |
| N11-1850 | Milk / Milk product | ST1290 | CC217 | 4b | I | Ebner *et al*., 2015 | QYIA00000000 | 3.5 |
| N12-1608 | human listeriosis | ST224 | CC224 | 1/2b | I | Althaus *et al*., 2014 | JABYZR000000000 | 3.5 |
| H34 | Human listeriosis | ST489 | CC489 | 1/2b | I | Muchaamba et al., 2018 | CP020774 | 3.5 |
| Lm10403S | Human listeriosis | ST85 | CC7 | 1/2a | II | Edman et al., 1968 | NC_017544 | 3.5 |
| N1546**^cd^** | Imported cooked ham | ST8 | CC8 | 1/2a | II | Hächler et al., 2013 | CP013724 | 4 |
| N11-1617 | Meat / Meat product | ST8 | CC8 | 1/2a | II | Ebner et al., 2015 | JABZDM000000000 | 4 |
| N11-1649 | Meat / Meat product | ST743 | CC8 | 1/2a | II | Ebner et al., 2015 | JABZDL000000000 | 3.5 |
| N11-1584 | human listeriosis | ST1295 | CC8 | 1/2a | II | Althaus et al., 2014 | JABZAJ000000000 | 3.5 |
| N11-1346 | Human listeriosis | ST673 | CC8 | 1/2a | II | Althaus *et al*., 2014 | JABZDQ000000000 | 3.5 |
| EGDe**^d^** | Rabbits | ST35 | CC9 | 1/2a | II | Glaser et al., 2001 | NC_003210.1 | 3.5 |
| D:824/5 | Meat product | ST9 | CC9 | 3c | II | Ebner et al., 2015 | NS | 4 |
| N11-1514 | Meat / Meat product | ST9 | CC9 | 1/2c | II | Ebner et al., 2015 | JABYZD000000000 | 3.5 |
| N12-1921 | Plant associated | ST9 | CC9 | 1/2c | II | Ebner et al., 2015 | JABYYW000000000 | 3.5 |
| D: 650/8 | Meat / Meat product | ST9 | CC9 | 3c | II | Ebner et al., 2015 | NS | 3.5 |
| N12-0152 | Milk / Milk product | ST9 | CC9 | 1/2a | II | Ebner et al., 2015 | NS | 3.5 |
| N11-1837 | human listeriosis | ST9 | CC9 | 1/2a | II | Althaus et al., 2014 | JABZBE000000000 | 3.5 |
| N12-0486 | human listeriosis | ST9 | CC9 | 1/2c | II | Althaus et al., 2014 | JABYZC000000000 | 3.5 |
| N13-0001 | human listeriosis | ST9 | CC9 | 1/2c | II | Althaus et al., 2014 | JABYYS000000000 | 3.5 |
| N12-1864 | Milk / Milk product | ST9 | CC9 | 1/2a | II | Ebner et al., 2015 | NS | 3.5 |
| Lm3136**^c^** | Tomme cheese | ST18 | CC18 | 1/2a | II | Bille et al., 2006 | CP013723 | 4 |
| N11-2183 | Plant associated | ST20 | CC20 | 1/2a | II | Ebner et al., 2015 | JABZDF000000000 | 3.5 |
| Lm3163**^c^** | Tomme cheese | ST26 | CC26 | 1/2a | II | Bille et al., 2006 | CP013722 | 3.5 |
| N11-1515 | Milk product | ST29 | CC29 | 1/2a | II | Ebner et al., 2015 | JABZDO000000000 | 4 |
| N05-195**^f^** | Meat / Meat product | ST31 | CC31 | 1/2a | II | Ebner et al., 2015 | QYIT00000000 | 4 |
| N11-1905 | Meat / Meat product | ST121 | CC121 | 1/2a | II | Ebner *et al*., 2015 | JABZDG000000000 | >4 |
| N12-1024 | Meat / Meat product | ST121 | CC121 | 1/2a | II | Ebner *et al*., 2015 | JABZCE000000000 | 3.5 |
| N13-0119 | human listeriosis | ST121 | CC121 | 1/2a | II | Althaus *et al*., 2014 | JABZAK000000000 | >4 |
| N12-0367 | human listeriosis | ST121 | CC121 | 1/2a | II | Althaus *et al*., 2014 | JABZAE000000000 | >4 |
| N842_15 | Human prosthetic joint | ST412 | CC412 | 1/2a | II | Muchaamba et al., 2020 | CP046361 | 3 |
| N843_10 | Human prosthetic joint | ST412 | CC412 | 1/2a | II | Muchaamba et al., 2020 | CP046362 | 3.5 |
| N12-1273 | Human listeriosis | ST412 | CC412 | 1/2a | II | Althaus *et al*., 2014 | QYFZ00000000 | 3.5 |
| WSLC1019 | Animal isolate | ST130 | CC69 | 4c | III | ATCC 19116 | CP013286 | 3.5 |
| LMNC318**^d^** | Ruminant listeriosis | ST70 | CC70 | 4a/4c | III | Dreyer et al., 2016 | ERS1324346 | 4 |
| LMNC326**^d^** | Ruminant listeriosis | ST70 | CC70 | 4a/4c | III | Dreyer et al., 2016 | ERS1324347 | 4 |
| WLSC1020 | Animal isolate | ST71 | CC131 | 4a | III | ATCC 19114 | NZ_CP013287 | 3.5 |
| F2365 | Genome control | ST1 | CC1 | 4b | I | Nelson et al., 2004 | NC_002973 |  |
| JF5051 | *L. innocua* control |  |  |  |  | Guldimann et al., 2015 | NS |  |

**^a^**ST: sequence type; CC: clonal complex.  **^b^** Strains with MIC ≥ 4 µg/mL are classified as BC stress resistant. **^c^**Listeriosis outbreak strains. **^d^**Strains used for tracking bacteria growth within zebrafish embryos. **^e^**Strains used in the dual osmotic plus cold stress experiments. **NS**: not sequenced. **^f^**Also known as N14-195.

# References

Althaus D, Lehner A, Brisse S, Maury M, Tasara T, and Stephan R. 2014. Characterization of *Listeria monocytogenes* strains isolated during 2011-2013 from human infections in Switzerland. Foodborne. Pathog. Dis; 11: 753-758. doi: 10.1089/fpd.2014.1747.

Althaus, D.; Jermini, M.; Giannini, P.; Martinetti, G.; Reinholz, D.; Nüesch-Inderbinen, M.; Lehner, A.; Stephan, R. Local Outbreak of *Listeria monocytogenes* Serotype 4b Sequence Type 6 due to Contaminated Meat Pâté. Foodborne Pathog. Dis. 2017, 14, 219–222.

Bille J. 1990. Epidemiology of human listeriosis in Europe with special reference to the Swiss outbreak, p 71-74 In Miller AJ, Smith JL, Somkuti GA, editors. Foodborne listeriosis. Elsevier, New York.

Bille, J.; Blanc, D.S.; Schmid, H.; Boubaker, K.; Baumgartner, A.; Siegrist, H.H.; Tritten, M.L.; Lienhard, R.; Berner, D.; Anderau, R.; et al. Outbreak of human listeriosis associated with tomme cheese in northwest Switzerland, 2005. Eurosurveillance 2006, 11, 11–12.

Dreyer, M.; Aguilar-Bultet, L.; Rupp, S.; Guldimann, C.; Stephan, R.; Schock, A.; Otter, A.; Schüpbach, G.; Brisse, S.; Lecuit, M.; et al. *Listeria monocytogenes* sequence type 1 is predominant in ruminant rhombencephalitis. Sci. Rep. 2016, 6, 36419.

Ebner R, Stephan R, Althaus D, Brisse S, Maury M, and Tasara T. 2015. Phenotypic and genotypic characteristics of *Listeria monocytogenes* strains isolated during 2011-2014 from different food matrices in Switzerland. Food Control; 57: 321-326. doi: 10.1016/j.foodcont.2015.04.030

Edman, D.C.; Pollock, M.B.; Hall, E.R. *Listeria monocytogenes* L Forms I. Induction, Maintenance, and Biological Characteristics1. J. Bacteriol. 1968, 96, 352–357.

Fleming, D.W.; Holmes, M.B.; Audurier, A.; Cochi, S.L.; Macdonald, K.L.; Brondum, J.; Hayes, P.S.; Plikaytis, B.D.; Broome, C.V.; Reingold, A.L. Pasteurized Milk as a Vehicle of Infection in an Outbreak of Listeriosis. N. Engl. J. Med. 1985, 312, 404–407.

Glaser, P.; Frangeul, L.; Buchrieser, C.; Rusniok, C.; Amend, A.; Baquero, F.; Berche, P.; Bloecker, H.; Brandt, P.; Chakraborty, T.; et al. Comparative genomics of *Listeria* species. Science 2001, 294, 849–852.

Guldimann C, Bärtschi1 M, Frey J, Zurbriggen A, Seuberlich T, and Oevermann A. 2015. Increased spread and replication efficiency of *Listeria monocytogenes* in organotypic brain-slices is related to multilocus variable number of tandem repeat analysis (MLVA) complex. BMC Microbiol; 15: 134. doi: 10.1186/s12866-015-0454-0.

Hächler, H.; Marti, G.; Giannini, P.; Lehner, A.; Jost, M.; Beck, J.; Weiss, F.; Bally, B.; Jermini, M.; Stephan, R.; et al. Outbreak of Listerosis due to Imported Cooked Ham, Switzerland 2011. Eurosurveillance 2013, 18. Available online: http://www.eurosurveillance.org/ViewArticle.aspx?ArticleId=20469 (accessed on 11 August 2020).

Muchaamba, F.; Eshwar, A.K.; Von Ah, U.; Stevens, M.J.A.; Tasara, T. Evolution of *Listeria monocytogenes* During a Persistent Human Prosthetic Hip Joint Infection. Front. Microbiol. 2020, 11, 1726

Muchaamba, F.; Guldimann, C.; Tasara, T.; Mota, M.I.; Braga, V.; Varela, G.; Algorta, G.; Klumpp, J.; Jermini, M.; Stephan, R. Full-Genome Sequence of *Listeria monocytogenes* Strain H34, Isolated from a Newborn with Sepsis in Uruguay. Genome Announc. 2017, 5, e00544-17.

Nelson KE, Fouts DE, Mongodin EF, Ravel J, DeBoy RT, Kolonay JF, Rasko DA, Angiuoli SV, Gill SR, et al. 2004. Whole genome comparisons of serotype 4b and 1/2a strains of the food-borne pathogen *Listeria monocytogenes* reveal new insights into the core genome components of this species. Nucleic Acids Res. Apr 28;32(8):2386-95. doi: 10.1093/nar/gkh562.

Stephan, R.; Althaus, D.; Kiefer, S.; Lehner, A.; Hatz, C.; Schmutz, C.; Jost, M.; Gerber, N.; Baumgartner, A.; Hächler, H.; et al. Foodborne transmission of *Listeria monocytogenes* via ready-to-eat salad: A nationwide outbreak in Switzerland, 2013–2014. Food Control 2015, 57, 14–17.

Tasara T, Ebner R, Klumpp J, Stephan R. 2015. Complete genome sequence of *Listeria monocytogenes* N2306, a strain associated with the 2013-2014 listeriosis outbreak in Switzerland. Genome Announc 3(3):e00553-15. doi:10.1128/genomeA.00553-15.

Tasara T, Klumpp J, Bille J, Stephan R. 2016. Genome sequences of *Listeria monocytogenes* strains responsible for cheese- and cooked ham product-associated Swiss listeriosis outbreaks in 2005 and 2011. Genome Announc 4(2):e00106-16. doi:10.1128/genomeA.00106-16.
